# Notch Signaling Regulates Late-Stage Epidermal Differentiation and Maintains Postnatal Hair Cycle Homeostasis

**DOI:** 10.1371/journal.pone.0015842

**Published:** 2011-01-18

**Authors:** Hsien-Yi Lin, Cheng-Heng Kao, Kurt Ming-Chao Lin, Vesa Kaartinen, Liang-Tung Yang

**Affiliations:** 1 Institute of Cellular and System Medicine, National Health Research Institutes, Miaoli County, Taiwan, Republic of China; 2 Center of General Education, Chang Gung University, Tao-Yuan, Taiwan, Republic of China; 3 Division of Medical Engineering, National Health Research Institutes, Miaoli County, Taiwan, Republic of China; 4 Department of Biologic and Materials Sciences, University of Michigan, Ann Arbor, Michigan, United States of America; Childrens Hospital Los Angeles, United States of America

## Abstract

**Background:**

Notch signaling involves ligand-receptor interactions through direct cell-cell contact. Multiple Notch receptors and ligands are expressed in the epidermis and hair follicles during embryonic development and the adult stage. Although Notch signaling plays an important role in regulating differentiation of the epidermis and hair follicles, it remains unclear how Notch signaling participates in late-stage epidermal differentiation and postnatal hair cycle homeostasis.

**Methodology and Principal Findings:**

We applied Cre/loxP system to generate conditional gene targeted mice that allow inactivation of critical components of Notch signaling pathway in the skin. *Rbpj*, the core component of all four Notch receptors, and *Pofut1*, an essential factor for ligand-receptor interactions, were inactivated in hair follicle lineages and suprabasal layer of the epidermis using the *Tgfb3*-Cre mouse line. *Rbpj* conditional inactivation resulted in granular parakeratosis and reactive epidermal hyperplasia. *Pofut1* conditional inactivation led to ultrastructural abnormalities in the granular layer and altered filaggrin processing in the epidermis, suggesting a perturbation of the granular layer differentiation. Disruption of *Pofut1* in hair follicle lineages resulted in aberrant telogen morphology, a decrease of bulge stem cell markers, and a concomitant increase of K14-positive keratinocytes in the isthmus of mutant hair follicles. *Pofut1*-deficent hair follicles displayed a delay in anagen re-entry and dysregulation of proliferation and apoptosis during the hair cycle transition. Moreover, increased DNA double stand breaks were detected in *Pofut1*-deficent hair follicles, and real time PCR analyses on bulge keratinocytes isolated by FACS revealed an induction of DNA damage response and a paucity of DNA repair machinery in mutant bulge keratinocytes.

**Significance:**

our data reveal a role for Notch signaling in regulating late-stage epidermal differentiation. Notch signaling is required for postnatal hair cycle homeostasis by maintaining proper proliferation and differentiation of hair follicle stem cells.

## Introduction

Skin epidermis serves as a barrier to prevent the loss of body fluid and separate the body from most environmental insults. The epidermis develops from a single layer of proliferative keratinocytes to multi-layered stratified epithelium consisting of a basal layer, spinous layer, granular layer, and stratum corneum. As basal keratinocytes move upward and differentiate along the way to the skin surface, they acquire layer-specific characteristics, including specialized epidermal keratins and cell junctions. Cells at the stratum corneum finally cease their metabolic activity and extrude lipid bilayers to build up the epidermal barrier. As the epidermis undergoes stratification, it also generates appendages, such as hair follicles and associated sebaceous glands or sweat glands [1]. After the initial morphogenesis, hair follicles undergo repeated cycles of catagen (regression phase), telogen (resting phase), and anagen (proliferation phase) throughout the adult lifetime. This homeostasis is maintained by quiescent hair follicle stem cells residing in the bulge region located below the sebaceous glands [2]. In addition to their ability to regenerate hair follicle cell lineages, hair follicle stem cells can repair damaged epidermis under stressed conditions, such as wounding [3], [4].

In mammals, four Notch receptors (Notch1–4) and five canonical Notch ligands (Jagged1–2, Delta-like1, 3, and 4) have been identified. Ligand-receptor interactions between contacting cells lead to conformational changes in the Notch receptor, which facilitate sequential proteolysis, first by ADAM family members and followed by γ-secretase/presenilins, to generate Notch intracellular domain (NICD). NICD translocates into the nucleus and binds to Rbpj and Mastermind, thereby activating the transcription of target genes, e.g. members of the *Hes* and *Hey* family [5]. Notch signaling is modulated by glycosylation of the extracellular domain of Notch receptors [6]. One of the modifiers is protein O-fucosyltransferase 1 (*Pofut1*), which transfers O-fucose to a particular consensus sequence in the EGF-like repeats of Notch receptor extracellular domain [7] and is ubiquitously expressed in mammalian tissues [8]. Biochemical studies demonstrated that O-fucose modification of mammalian Notch receptors is required for efficient ligand-receptor binding and subsequent signal transduction [9]. Loss of *Pofut1* in the mouse embryo resulted in a severe phenotype similar to that of embryos lacking core components of Notch signaling pathway, such as *Presenilins* and *Rbpj* [10], [11], [12].

Multiple Notch receptors and ligands are expressed in the epidermis and hair follicles during embryonic development and the adult stage [13]. Loss-of-function and gain-of-function studies in cell culture and animal models have demonstrated that Notch signaling regulates early-stage differentiation of the epidermis [14], [15], [16]. A role for Notch signaling in promoting granular layer differentiation has been suggested by *in vitro* studies of human keratinocytes using an agonist peptide [17], and by transgenic expression of NICD in the suprabasal layer of the epidermis using the *involucrin* promoter [18]. Loss of Notch signaling does not affect hair follicle patterning or hair placode formation; however, Notch signaling is required for complete maturation of hair follicles [15], [19]. While Notch signaling has a significant role in regulating differentiation of the epidermis and hair follicles, it remains unclear how Notch signaling participates in late-stage epidermal differentiation and postnatal hair cycle homeostasis. Interestingly, epithelial deletion of Notch1 results in a shortened anagen period and premature entry into catagen at the first hair cycle, suggesting that Notch1 is involved in hair cycle regulation [20].

**Figure 1:**
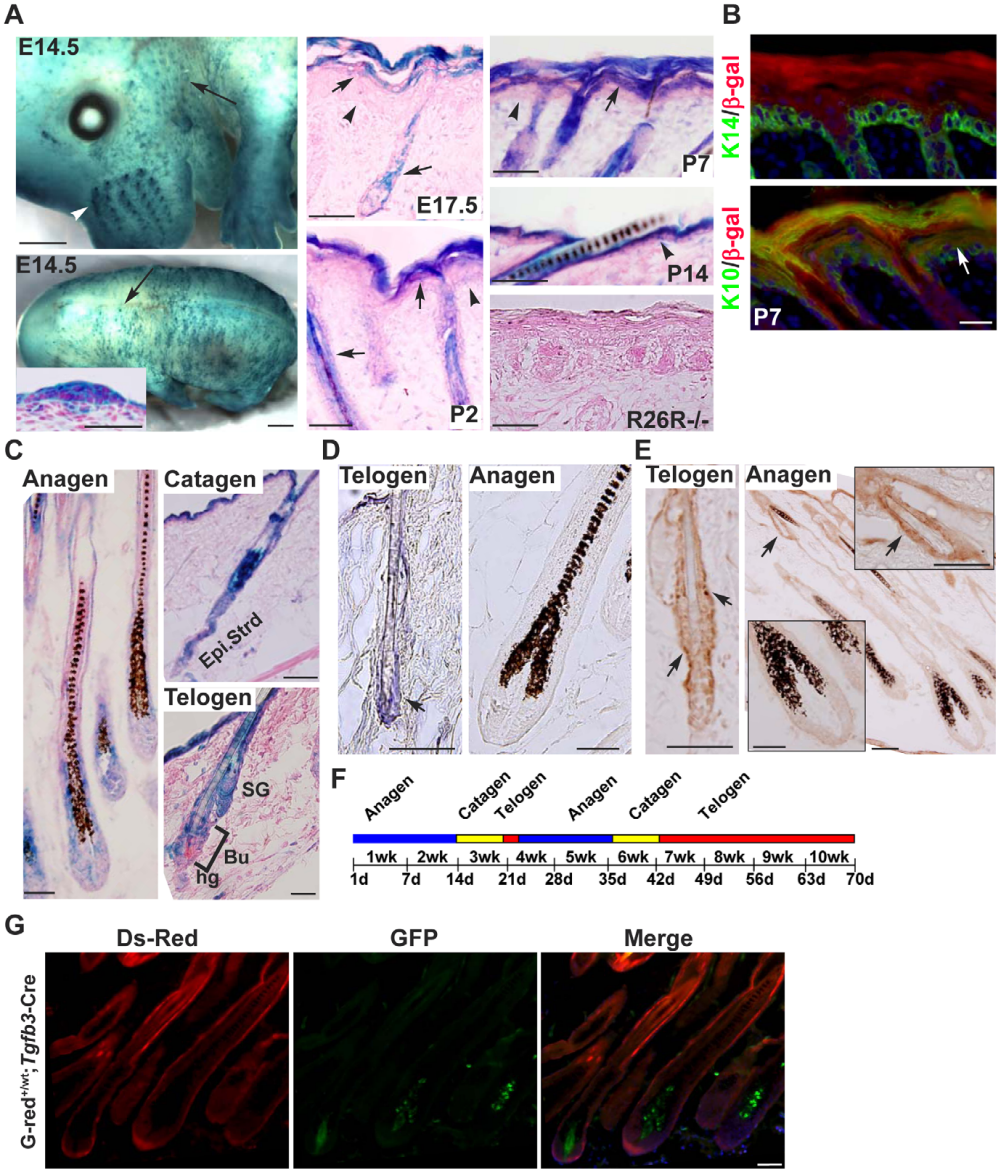
Analysis of the *Tgfb3*-Cre-induced recombination in the back skin using Rosa26 and G-red reporter mice. (**A**) X-gal-stained E14.5 embryos and back skin sagittal sections from E14.5 to P14. The inset shows a *LacZ*-positive hair placode from a cross section of the trunk. The arrowhead denotes a junction between the epidermis and dermis. Back skin sections of E17.5 R26R−/− mice did not display any *lacZ*-positive signals. (**B**) Back skin sagittal sections at P7 were double-stained for K14 (green) and β-galactosidase (red), and for K10 (green) and β-gal (red). Upper K10 layer stained positive for β-gal (arrow). (**C**) X-gal-stained back skin sections at different stages of the hair cycle. Epi. Strd: epithelium strand, Bu: Bulge, hg: hair germ. (**D**) *In situ* hybridization of *Tgfb3* on back skin sections at telogen and anagen. (**E**) Immunostaining for Cre on back skin samples from R26R^+/wt^; *Tgfb3*-Cre^+/wt^ mice at telogen and anagen. Positive nuclear staining (brown color) was indicated by arrows. Insets show bulge and bulb region at high magnification. (**F**) Illustration of the first and second hair cycle of mice corresponding to their age. (**G**) Back skin sagittal sections of G-red^+/wt^;*Tgfb3*-Cre^+/wt^ mice at P30 were examined by fluorescent microscopy. Ds-Red-positive (Red) and GFP-positive (green) signals are shown in separate panels and superimposed image (Merge) is shown in the right panel. Scale bar, 50 µM.

Three *Tgfb* isoforms (*Tgfb1*, *Tgfb2*, and *Tgfb3*) are expressed in the epidermis and hair follicle during the embryonic and adult stage [21], [22]. We previously made a *Tgfb3*-Cre mouse line for palatal epithelium studies [23], and we also found that the *Tgfb3* promoter-driven Cre induces recombination in the suprabasal layer of the epidermis and hair follicle epithelium including the bulge region (Figure 1). Since grafted skin from *Tgfb3* null mice does not display any defect in epidermal or hair follicle development [24], the *Tgfb3*-Cre mouse line was used in our study to investigate the role of Notch signaling in terminal differentiation of the epidermis and hair cycle homeostasis. We applied two approaches to disable distinct mechanisms of Notch signaling. First, *Rbpj*, the core component of all four Notch receptors, was deleted to block Notch signaling at the transcriptional events. Second, *Pofut1*, an essential factor for ligand-receptor interactions, was deleted to block Notch signaling upstream of transcriptional events. We found that canonical Notch signaling is required for late-stage epidermal differentiation and correct processing of filaggrin, a process when perturbed being closely associated with barrier function defects and skin disorders. *Pofut1* deletion in hair follicle lineages resulted in a decrease of hair follicle stem cell markers and an increase of K14-expressing keratinocytes in the isthmus. The mutant hair follicles displayed a delay in anagen re-entry and dysregulation of proliferation and apoptosis during the hair cycle transition, which may be caused by DNA damage response and downregulation of DNA repair genes in hair follicle stem cells.

## Results

### *Tgfb3*-Cre induces recombination in epidermal and hair follicle lineages

We used Rosa26 mouse as a reporter to detect *Tgfb3*-Cre-induced recombination during embryonic and postnatal epidermal development, and the resulting *Tgfb3*-Cre^+/wt^;R26R^+/−^ mice at different developmental stages were analyzed for β-galactosidase activity. At E14.5, whole mount *lacZ* staining revealed positive staining in whisker follicles (Figure 1A, E14.5, white arrowhead) and hair placode-like structures (Figure 1A, E14.5, arrows and inset). In addition, X-gal staining of back skin sections at E17.5 and P2 revealed a non-continuous staining in the granular layer and stratum corneum as well as in downward-growing hair follicles (arrows). At P7, the patchy *lacZ*-positive staining pattern in the epidermis became continuous. Double staining for β-galactosidase (β-gal) and K14, as well as for β-gal and K10 also revealed that *Tgfb3*-Cre recombines the reporter allele in the upper K10-expressing cell layers (Figure 1B). At P14, positive staining of *lacZ* remained at the suprabasal layer with the underneath displaying a speckled staining pattern. Next, *Tgfb3*-Cre-induced recombination in hair follicles at different hair cycle stages (Figure 1F) were examined by X-gal staining (Figure 1C). In anagen, positive staining was detected in follicular lineages. In catagen, *lacZ* staining was observed in the regressing epithelial strand and around the bulge region. In telogen, the bulge region, sebaceous glands, and infundibulum stained positive for *lacZ*.

We used *In situ* hybridization of *Tgfb3* (Figure 1D), immunostaining for Cre (Figure 1E), and G-red reporter mice (Figure 1G) to validate the recombination pattern. In telogen, *Tgfb3* mRNA transcripts were detected in the bulge region, sebaceous glands, and infundibulum. In anagen, *Tgfb3* mRNA transcript was under the detection level in the bulb region, which is a different finding from that of the Rosa26 reporter assay. Examination of Cre protein expression on back skin sections revealed that Cre is expressed in the bulge epithelium but not in the hair bulb, suggesting that *lacZ-*positive hair bulb matrix cells were likely derived from bulge cells that experienced Cre-induced recombination. Using G-red allele, another reporter which switches from GFP to Ds-Red expression upon Cre-induced recombination, we found that *Tgfb3*-Cre induces recombination in follicular lineages but not in the dermal papilla. Altogether, our results indicated that *Tgfb3*-Cre induces recombination in hair follicle cell lineages and in the suprabasal layer of the epidermis.

### *Rbpj* deletion by *Tgfb3*-Cre resulted in subtle defect in the granular layer and reactive epidermal hyperplasia

**Figure 2:**
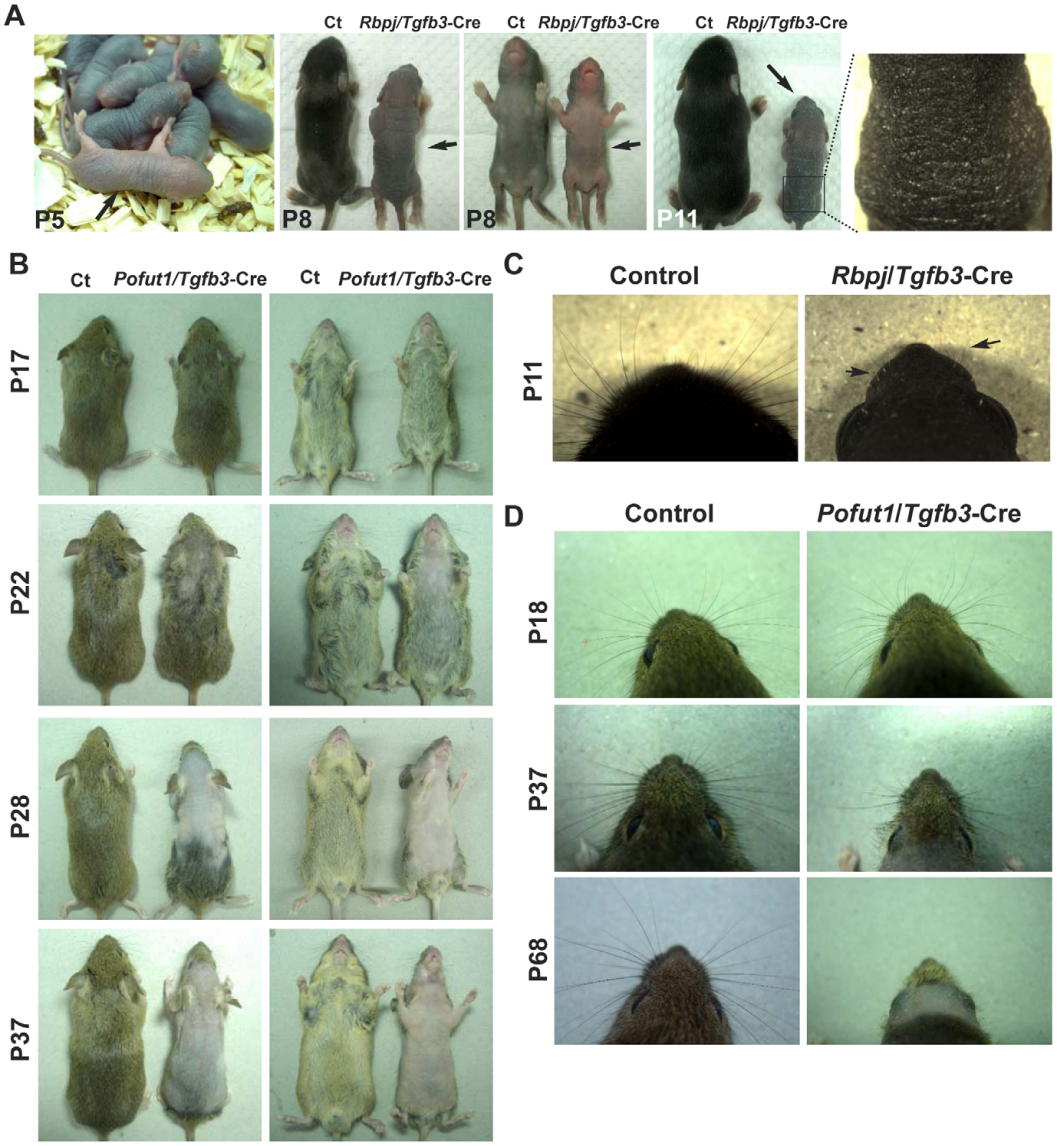
Gross appearance of *Rbpj*/*Tgfb3*-Cre and *Pofut1*/*Tgfb3*-Cre conditional knockout mice. (**A**) Comparison of the pelage from control and *Rbpj*/*Tgfb3*-Cre mice at the age of 5 days (P5), 8 days (P8), and 11 days (P11). The mutant mice were indistinguishable from their littermate control mice before P5. After P5, the mutant mice (arrows) were growth-retarded and did not develop hair coat as compared to their littermate controls (Ct). A magnified view of the back skin of *Rbpj*/*Tgfb*3-Cre mice at P11 showing severely dry and scaled skin. (**B**) Comparison of the pelage from *Pofut1*/*Tgfb3*-Cre mice and their littermate control mice at P17, P22, P28, and P37. Notably, the bald phenotype progresses in a head-to-tail direction. (**C**) Comparison of the whiskers from control and *Rbpj*/*Tgfb3*-Cre mice at P11. Whiskers of the *Rbpj*/*Tgfb3*-Cre mice were short and rudimentary. (**D**) Comparison of the whiskers from control and *Pofut1*/*Tgfb3*-Cre mice at P18, P37, and P68. *Pofut1*/*Tgfb3*-Cre mice gradually lose their whiskers.

To investigate the role of Notch signaling in late-stage epidermal differentiation and hair cycle homeostasis, *Pofut1* and *Rbpj* were inactivated by *Tgfb3*-Cre. Both *Rbpj* ^fx/fx^;*Tgfb3*-Cre^+/wt^ (*Rbpj*/*Tgfb3*-Cre) and *Pofut1* ^fx/fx^;*Tgfb3*-Cre^+/wt^ (*Pofut1*/*Tgfb3*-Cre) mice were born without overt abnormalities when compared to their littermate controls. *Rbpj*/*Tgfb3*-Cre mice did not develop pelage after P5 and later displayed severely dry and scaled skin at P11 (Figure 2A). They also had very short whiskers compared to their control littermates at P11 (Figure 2C). *Rbpj*/*Tgfb3*-Cre mice were growth-retarded from P5 onward and died of undetermined causes within two weeks after birth. In contrast, *Pofut1*/*Tgfb3*-Cre mice had an average life span of 4–5 months and exhibited progressive alopecia in a head-to-tail direction after 3 weeks postpartum (Figure 2B). The pelage hairs of *Pofut1*/*Tgfb3*-Cre mice sometimes grew back during the fifth week of life, but they were thinly scattered and short. *Pofut1*/*Tgfb3*-Cre mice gradually lost their whiskers by 10 weeks postpartum (Figure 2D). Interestingly, *Pofut1*/*Tgfb3*-Cre mice displayed splenomegaly and lymphoadenopathy around 3 weeks of age and developed full-blown atopic dermatitis-like disease in adult (data not shown). Mice harboring a conditional single *Pofut1* or *Rbpj* deletion were indistinguishable from their littermate controls regarding hair follicle and epidermal development (n>3, data not shown). A detailed analysis of other postnatal phenotypes is beyond the scope of this paper and will be described elsewhere.

**Figure 3:**
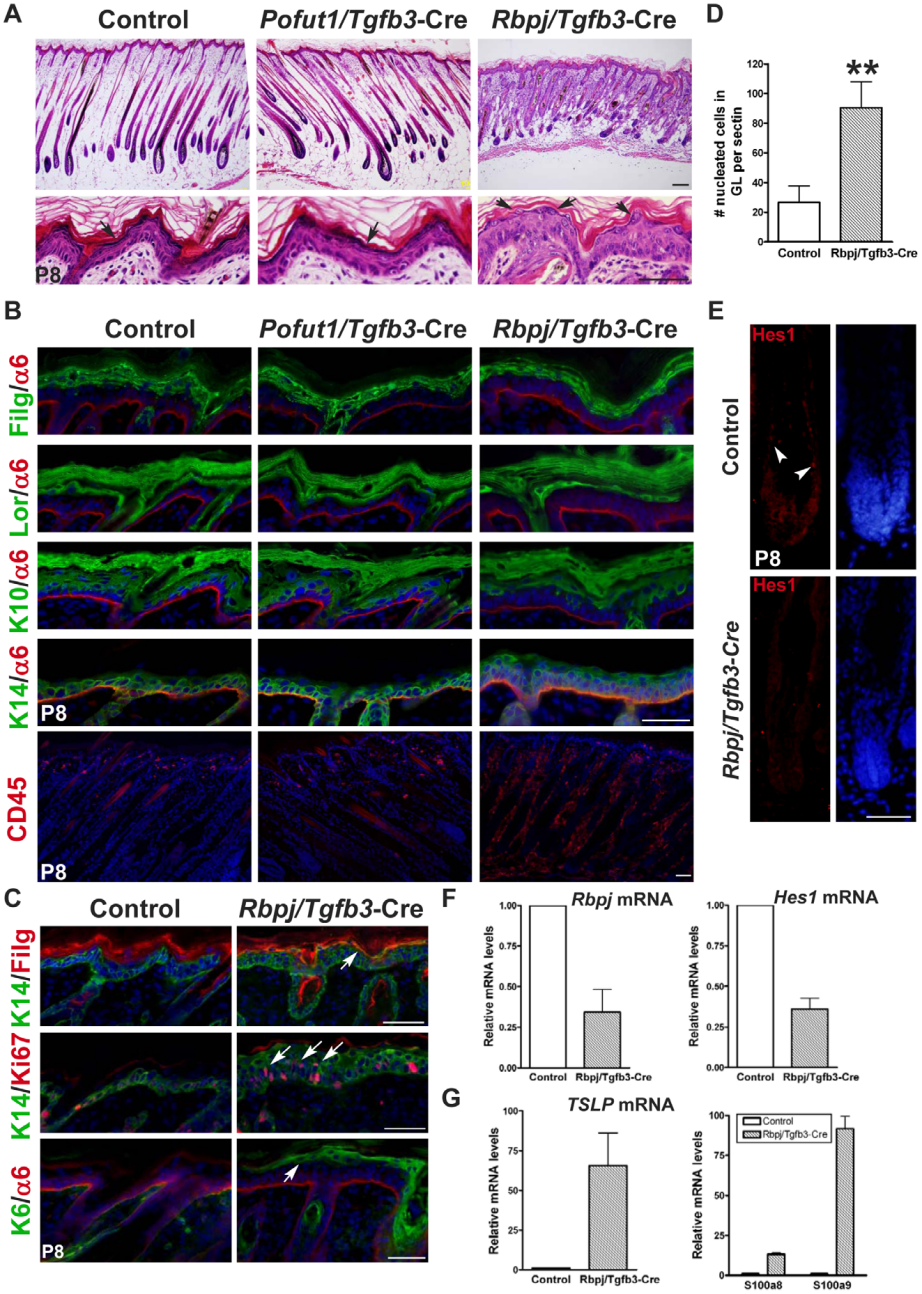
*Rbpj*/*Tgfb3*-Cre mice display an earlier onset of epidermis and hair follicle defects when compared with *Pofut1*/*Tgfb3*-Cre mice. (**A**) Low magnification (upper panels) and high magnification (lower panels) images of H&E stained back skin sagittal sections from age-matched control, *Pofut1*/*Tgfb3*-Cre, and *Rbpj*/*Tgfb*3-Cre mice at P8. Notably, the *Rbpj*/*Tgfb*3-Cre skin displayed acanthosis and granular parakeratosis (arrows). (**B**) Back skin samples from control, *Pofut1*/*Tgfb3*-Cre, and *Rbpj*/*Tgfb3*-Cre mice at P8 were analyzed for epidermal keratin and pan-leukocyte markers. The *Rbpj*/*Tgfb*3-Cre skin displayed a thickened K14-expressing layer and a massive infiltration of CD45+ leucocytes when compared with aged-match control and *Pofut1*/*Tgfb3*-Cre skin. α6 integrin (α6) labeled the basement membrane. (**C**) Back skin sagittal sections of control and *Rbpj*/*Tgfb*3-Cre mice were double-stained for K14 and filaggrin (K14/Filg), K14 and Ki67 (K14/Ki67), and K6 and α6-integrin (K6/α6). (**D**) Quantification of nucleated cells in the outermost granular layer of control and *Rbpj*/*Tgfb3*-Cre epidermis at P8. A bar diagram shows the number of nucleated cells per section (mean+/−s.d., n = 6) from three independent control and mutant pairs. **: P<0.01. (**E**) Immunostaining for Hes1 on back skin samples from control and *Rbpj*/*Tgfb3*-Cre mice at P8. Hes1-positive (red, arrowhead) and DAPI-counterstained (blue) signals are shown in separate panels. (**F, G**) qRT-PCR analysis of *Rbpj* and *Hes1* (F), TSLP, S100a8, and S100a9 (G) on back skin epithelium of control and *Rbpj*/*Tgfb3*-Cre mice at P8. Bar diagrams show mRNA levels of genes indicated in *Rbpj*/*Tgfb3*-Cre epidermis relative to corresponding controls (mean+/−s.d., n = 3, three independent control and mutant pairs). Antibodies used are color-coded according to fluorophore-tagged secondary antibodies. DAPI counterstaining in blue. Scale bar, 50 µM, except upper panels of (A), 100 µM.

Back skin samples from control, *Pofut1*/*Tgfb3*-Cre, and *Rbpj*/*Tgfb3*-Cre mice were further investigated by histological and immunofluorescence analyses at P8. At this time point the epidermis is still thick, allowing us to identify defects in epidermal stratification (Figure 3). H&E staining revealed that *Rbpj*/*Tgfb3*-Cre mice have epidermal hyperplasia and deteriorated hair follicles at P8 (Figure 3A). The epidermis of control and *Pofut1*/*Tgfb3*-Cre mice displayed proper stratification, while that of *Rbpj*/*Tgfb3*-Cre mice exhibited acanthosis and granular parakeratosis, as revealed by increased nucleated cells in the outermost granular layer of *Rbpj*/*Tgfb3*-Cre epidermis (Figure 3D). We found no differences in the expression levels of K10, loricrin, and filaggrin among control, *Pofut1*/*Tgfb3*-Cre, and *Rbpj*/*Tgfb3*-Cre mice, while an expansion of K14-expressing cell layers (Figure 3B) and an increase of Ki67-positive signal in the suprabasal K14-expressing keratinocytes (Figure 3C) was detected in the *Rbpj*/*Tgfb3*-Cre epidermis, indicating a hyperproliferative state. Moreover, the *Rbpj*/*Tgfb3*-Cre epidermis exhibited partial overlapping of K14 and filaggrin as well as ectopic K6 expression in the suprabasal keratinocytes, indicating premature differentiation and dysplasia, respectively (Figure 3C). Significantly, *Rbpj*/*Tgfb3*-Cre mice had increased CD45 staining in the dermis and subcutis than control and *Pofut1*/*Tgfb3*-Cre mice at P8, indicating inflammatory infiltrates (Figure 3B).

Epidermal hyperproliferation and acanthosis are typical compensatory responses of postnatal epidermis to compromised barrier function. Indeed, we observed increased expression levels of thymic stromal lymphopoietin (TSLP), a biomarker for the barrier function defect, and antimicrobial peptides (S100a8 and S100a9) in *Rbpj*/*Tgfb3*-Cre epidermis compared with control epidermis at P8 (Figure 3G). These data suggest that the inflammatory response seen in *Rbpj*/*Tgfb3*-Cre skin was due to defective barrier function. To determine whether the reactive epidermal hyperplasia was caused by a defect in epidermal stratification at an earlier time, histological and immunohistochemical studies were applied on control and *Rbpj*/*Tgfb3*-Cre epidermis at P1. (Figure S2). We found no differences in 1) the thickness of the epidermis, 2) expression levels of layer-specific epidermal markers, 3) the lipid deposit in the stratum corneum, and 4) outside-in permeability barrier function between control and *Rbpj*/*Tgfb3*-Cre epidermis. Collectively, our data indicate that the barrier function defect seen in the *Rbpj*/*Tgfb3*-Cre epidermis is not caused by impairment of epidermal stratification but rather due to intrinsic defect in the granular layer.

To confirm that the epidermis/hair follicle defects seen in *Rbpj*/*Tgfb3*-Cre mice were resulted from inactivation of Notch signaling, back skin samples from control and *Rbpj*/*Tgfb3*-Cre mice at P8 were analyzed for gene expression of *Rbpj* and *Hes1* using qRT-PCR (Figure 3F). We observed a decrease of *Rbpj* (∼66%) and *Hes1* (∼64%) mRNA levels in the *Rbpj*/*Tgfb3*-Cre epidermis relative to littermate controls. In addition, immunostaining of Hes1 on back skin sections revealed a loss of staining in *Rbpj*/*Tgfb3*-Cre hair follicles at P8, indicating a disruption of Notch signaling in the follicular lineages (Figure 3E). Oil red O staining and immunostaining of AE13 revealed sebaceous glands atrophy and hair shaft maturation defect, respectively, in *Rbpj*/*Tgfb3*-Cre hair follicles at P8 (Figure S1A, B), which is consistent with previous findings regarding *Rbpj* loss in hair follicle lineages [15]. Moreover, ectopic expression of K10 in degenerated hair follicles, a hallmark of *Rbpj* deletion in the dermal papilla [25], was not detected in *Rbpj*/*Tgfb3*-Cre mice at P8 (Figure S1, C), suggesting that the phenotype seen in mutants is less likely due to Notch signaling loss in the dermal papilla.

### *Pofut1* deletion in hair follicle lineages leads to aberrant telogen morphology while sebaceous gland homeostasis is less affected

**Figure 4:**
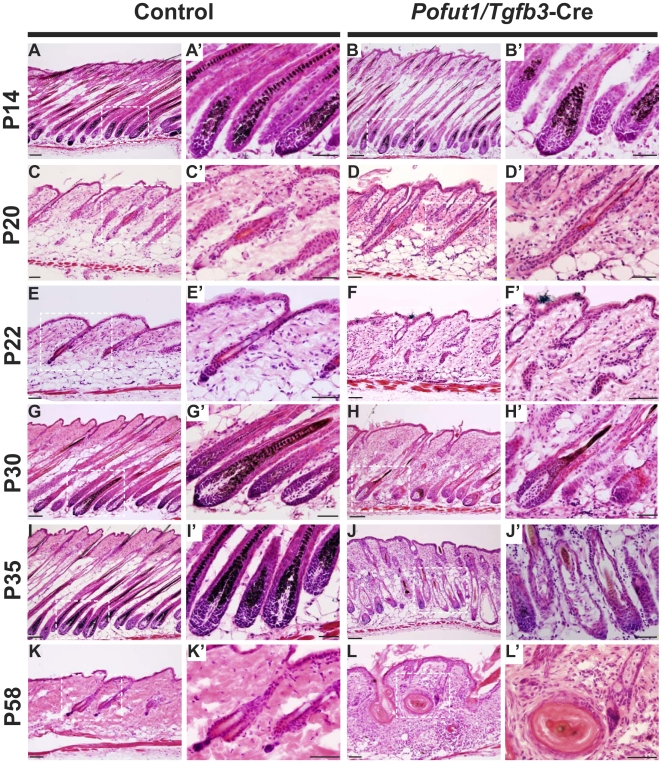
*Pofut1*/*Tgfb3*-Cre hair follicles display aberrant telogen morphology and have defects in hair follicle maturation. H&E stained back skin sections from control littermates (A, C, E, G, I, K) and *Pofut1*/*Tgfb3*-Cre mice (B, D, F, H, J, L) at P14 (first anagen), P20 (first catagen), P22 (first telogen), P30 (second anagen), P35 (second anagen), and P58 (second telogen). The boxed regions in (A–L) are shown at high magnification in the corresponding right panel (A′–L′). Hair follicles of *Pofut1*/*Tgfb3*-Cre mice displayed impaired differentiation (H) and subsequently turned into empty shells filled with keratinized materials (J). Most of the mutant hair follicles were converted to epithelial cysts at the end of the second hair cycle (L). The epidermis of *Pofut1*/*Tgfb3*-Cre mice exhibited progressive hyperplasia and hyperkeratosis. Scale bar, 100 µM in A, B, G, H, I, J; 50 µM in the rest of the panels.

Since *Pofut1*/*Tgfb3*-Cre mice displayed alopecia at the second hair cycle, we undertook a histological analysis on control and mutant mice at different stages of the hair cycle (Figure 4). During the first anagen (P14, Figure 4A, B), no obvious defects were observed in control littermates or mutant mice, and there was no accelerated catagen onset seen in mutant hair follicles (P20, Figure 4C, D). At the first postnatal telogen (P21–22, Figure 4E, F), control hair follicles exhibited well-defined bulge regions containing two cell layers of keratinocytes and were located below the sebaceous glands. In contrast, mutant hair follicles in position-matched sections displayed disorganized bulge regions with involuted epithelium and dispersed sebaceous glands, suggesting a failure to attain telogen morphology. Mutant hair follicles failed to complete hair shaft differentiation in the second hair cycle (Figure 4G–L), which is similar to the phenotype seen with compound deletion of Notch receptors in hair follicle lineages [26].

**Figure 5:**
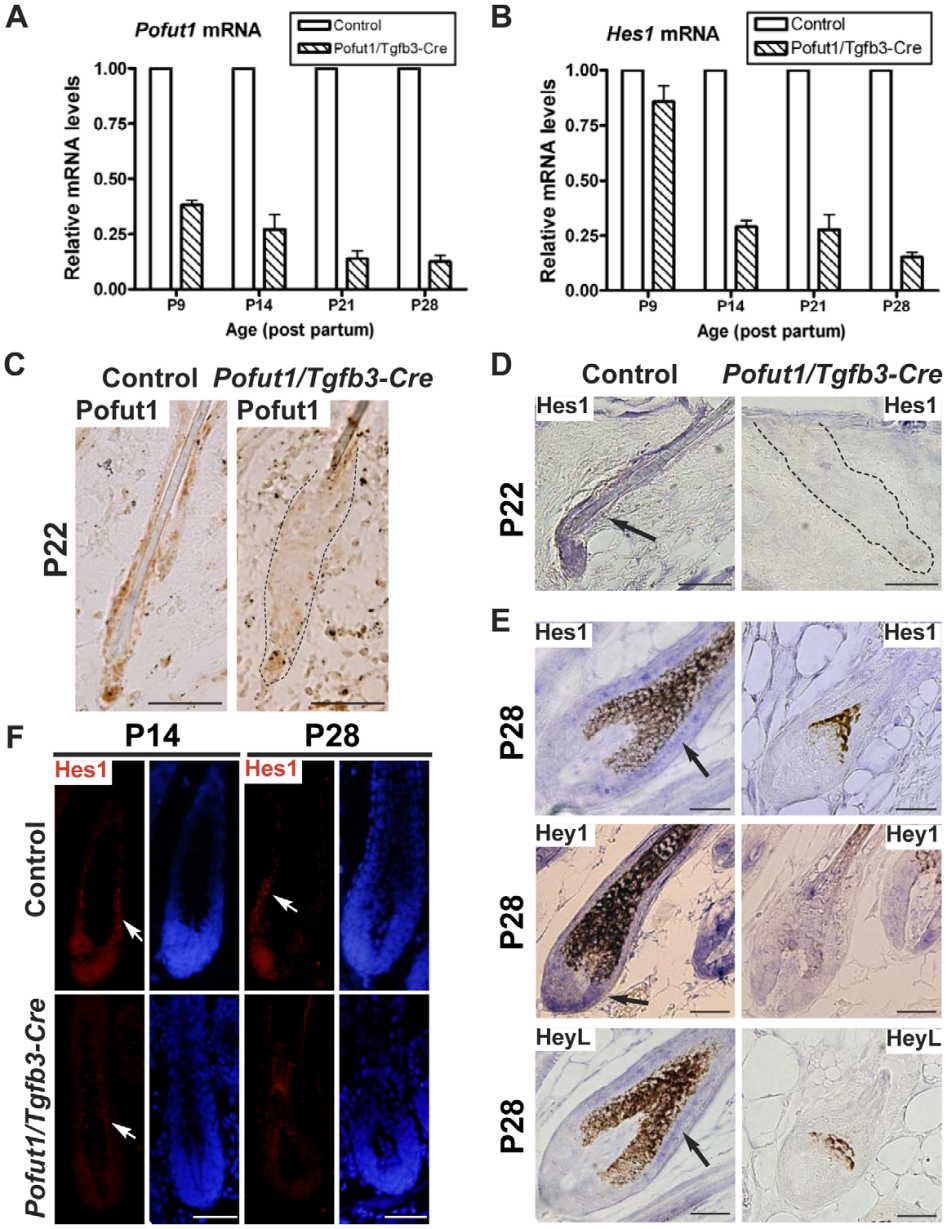
Conditional deletion of *Pofut1* and abrogation of Notch target genes in the *Pofut1*/*Tgfb3*-Cre skin. (**A, B**) qRT-PCR analysis of *Pofut1* and *Hes1* on back skin epithelium of control and *Pofut1*/*Tgfb3*-Cre mice at different time points. Bar diagrams show mRNA levels of *Pofut1* (A) and *Hes1* (B) in *Pofut1*/*Tgfb3*-Cre epidermis relative to corresponding controls (mean+/−s.d., n = 3, three independent control and mutant pairs per stage). (**C**) Immunohistochemical staining for Pofut1 on back skin samples from control and *Pofut1*/*Tgfb3*-Cre mice at P22. dotted line: a junction between the hair follicle and dermis. (**D, E**) *In situ* hybridization of Notch target genes on back skin samples from control and *Pofut1*/*Tgfb3*-Cre mice at P22 (D) and P28 (E). *Hes1* and *HeyL* are expressed in the precortex and inner root sheath, and *Hey1* is expressed in the hair bulb of control hair follicles. (**F**) Immunostaining for Hes1 on back skin samples from control and *Pofut1*/*Tgfb3*-Cre mice at P14 and P28. Hes1-positive (red, arrow) and DAPI-counterstained (blue) signals are shown in separate panels. Scale bar, 50 µM.

To confirm Notch signaling inactivation by *Pofut1* deletion, we analyzed back skin samples from control and *Pofut1*/*Tgfb3*-Cre mice for gene expression of *Pofut1* and Notch target genes. We observed a progressive decrease of *Pofut1* (from ∼60% to ∼90%) and *Hes1* (from ∼15% to ∼85%) mRNA levels in *Pofut1*/*Tgfb3*-Cre epidermis relative to control epidermis at intervals between P9 and P28 (Figure 5A, B). In addition, we found that Hes1 staining was only partially decreased in *Pofut1*/*Tgfb3*-Cre hair follicles at P14 and a complete loss of staining was observed at P28 (Figure 5F). Specific disruption of *Pofut1* and Notch signaling in *Pofut1*/*Tgfb3*-Cre hair follicles after P22 was demonstrated by immunostaining for *Pofut1* (Figure 5C) and by *in situ* hybridization of Hes1, Hey1, and HeyL (Figure 5D, E). Our data collectively suggested that conditional deletion of *Pofut1* by *Tgfb3*-Cre leads to a progressive inactivation of Notch signaling over time.

Next, Oil Red O staining was applied to investigate the effect of Notch signaling loss on sebaceous gland homeostasis (Figure S3). At P14, sebaceous glands of control and *Pofut1*/*Tgfb3*-Cre mice were comparable. After the first telogen (P22 to P35), we observed enlarged and disorganized sebaceous glands and sebum accumulation in the infundibulum of mutant hair follicles. By P58, mutant sebaceous glands were degenerated along with deteriorated hair follicles while the sebum could still be detected in the epithelial cysts. In summary, these results suggest that Notch signaling is not required for the homeostasis of sebaceous glands.

### Conditional deletion of *Pofut1* in the suprabasal layer of the epidermis perturbs the granular layer differentiation program

*Pofut1*/*Tgfb3*-Cre mice displayed hyperkeratosis and reactive hyperplasia with expanded K14-positive cell layers at P35 (Figure S4). Immunostaining of TSLP and qRT-PCR analysis of TSLP, S100a8, and S100a9 on back skin samples of control and *Pofut1*/*Tgfb3*-Cre mice at P14 revealed a greater increase of TSLP both at the mRNA and protein level, and a slight increase of S100a9 gene expression in the mutant epidermis compared with the control epidermis, suggestive of a barrier function defect (Figure S4D, E). Immunostaining for epidermal keratin markers and Nile red staining on control and mutant epidermis at P14 (Figure S4A, C) revealed that the barrier function defect is not due to impairment in epidermal stratification or lipid deposit.

**Figure 6:**
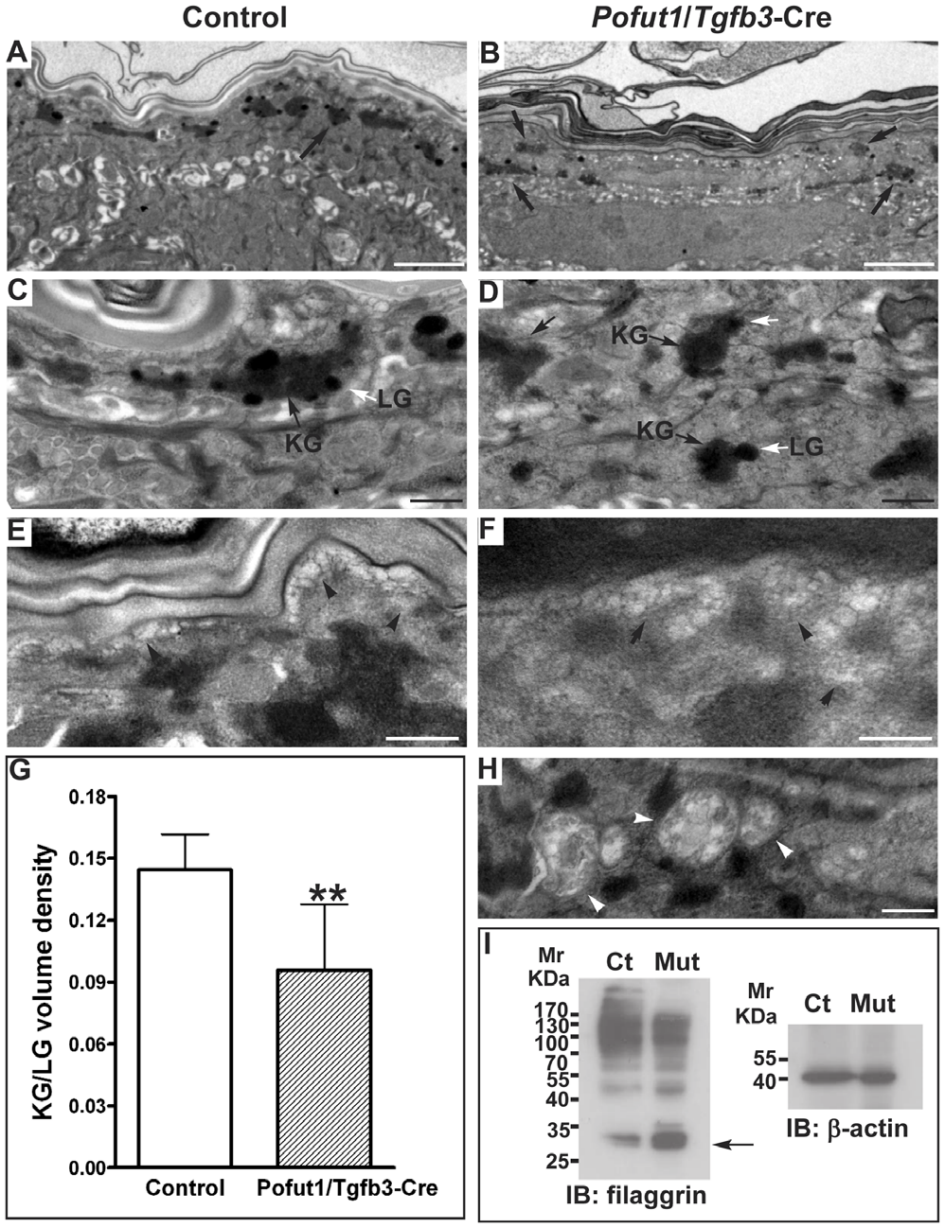
Abrogation of *Pofut1* in suprabasal keratinocytes leads to ultrastructural and biochemical changes in the granular layer. Ultrastructures of the epidermis from control littermates (A, C, E) and *Pofut1*/*Tgfb3*-Cre mice (B, D, F, H) at P17 were examined by transmission electron microscopy. (**A–D**) The keratohyalin granules (KG, arrows) and lamellar granules (LG, white arrows) are not correctly distributed in the granular layer. (**E, F**) Control and *Pofut1*/*Tgfb3*-Cre samples display normal exocytosis vesicles (arrowhead) at the granular layer/stratum corneum interface. (**G**) KG and LG are significantly reduced in the mutant epidermis. A bar diagram shows the volume density of KG/LG (mean+/−s.d.) from two independent control and mutant pairs, **:P<0.01. (**H**) Autophagosomes (white arrowheads) are observed in the granular layer of *Pofut1*/*Tgfb3*-Cre epidermis. Magnification: 6,000× in A, B; 30,000× in C–F and H. Scale bar: 2 µM, A and B; 0.5 µM, C–F and H. (**I**) Filaggrin immunoblots of isolated primary keratinocytes from control (Ct) and *Pofut1*/*Tgfb3*-Cre (Mut) mice at P17. β-actin immunoblots were used as loading control. Mr: protein standard molecular weight in kilodaltons (kDa). Arrow: monomeric filaggrin. Representative results from two independent control and mutant pairs.

To further investigate the cause of barrier function defect in *Pofut1*/*Tgfb3*-Cre epidermis, we applied transmission electron microscopy (TEM) to examine the late-stage epidermal differentiation in control and mutant mice (Figure 6). In the control epidermis, large keratohyalin granules (KGs) were distributed mainly in the uppermost granular keratinocytes (Figure 6A, C), and a few lamellar granules (LGs) could be seen in the underlying cell layer. In contrast, KGs were not restricted in the uppermost granular keratinocytes of the *Pofut1*/*Tgfb3*-Cre epidermis, but were also present in the lower cell layer (Figure 6B, D). KGs and LGs are significantly reduced in the mutant epidermis, as evidenced by a quantitative analysis of the volume density of KG/LG in control versus mutant epidermis (Figure 6G). We did not observe any defect in the secretion of lamellar bodies in mutant mice, as exocytosis vesicles were both present at the granular layer/stratum corneum interface in control and mutant epidermis (Figure 6E, F). Interestingly, a few autophagosomes were observed in the granular layer of mutant epidermis (Figure 6H).

The major component of the KGs is profilaggrin, and the processing of profilaggrin to filaggrin occurs in the KGs. We therefore analyzed filaggrin processing in control and *Pofut1*/*Tgfb3*-Cre epidermis by western blotting. The processing of profilaggrin to filaggrin was not different between control and mutant epidermis. However, we observed an increased level of monomeric filaggrin in the mutant epidermis than in control epidermis, suggesting an altered filaggrin processing occurring in the mutant epidermis (Figure 6I). Taken together, these data suggest a late-stage lesion in the granular layer of *Pofut1*/*Tgfb3*-Cre epidermis, which leads to a barrier function defect and subsequently induces reactive epidermal hyperplasia.

### Abrogation of *Pofut1* in hair follicle lineages alters bulge stem cell markers

**Figure 7:**
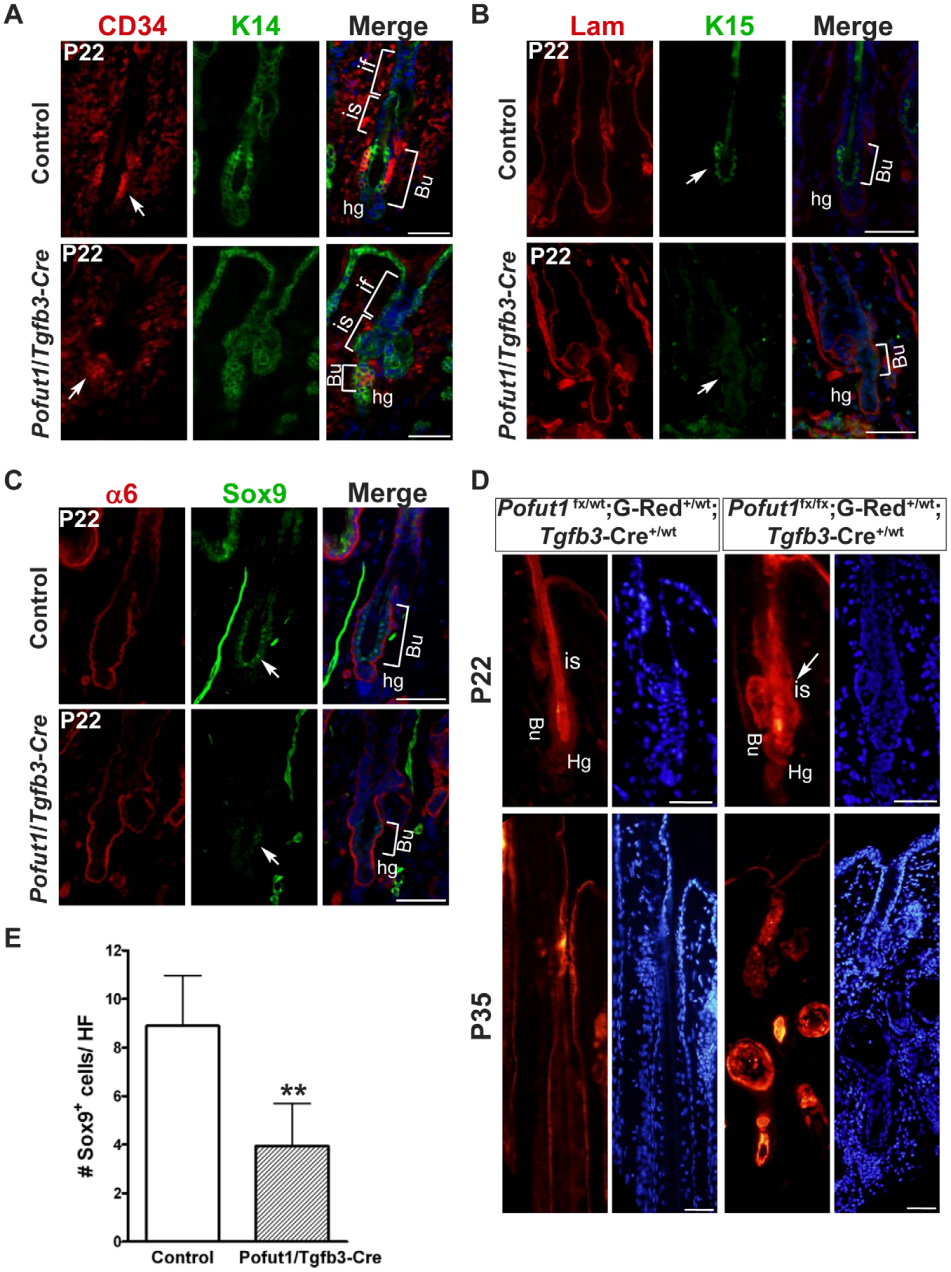
*Pofut1* deletion in hair follicle lineages has an impact on bulge stem cells. (**A**) Back skin samples from control and *Pofut1*/*Tgfb3*-Cre mice at P22 were double-stained for K14 and CD34. is: isthmus. if: infundibulum. Bu: bulge, hg: hair germ. (**B, C**) Back skin samples from control and *Pofut1*/*Tgfb3*-Cre mice were double-stained for K15 and laminin (K15/Lam) (B), and Sox9 and α6-integrin (Sox9/α6) (C). Images from individual staining are shown in separate panels and superimposed images are displayed in the corresponding right panel (Merge). Immunostaining for laminin or α6-integrin was used to confirm a proper orientation of the follicle in sections and misangled follicles were excluded from analyses. (**D**) Back skin samples from *Pofut1* ^fx/wt^;G-Red^+/wt^;*Tgfb3*-Cre^+/wt^ and *Pofut1* ^fx/fx^;G-Red^+/wt^;*Tgfb3*-Cre^+/wt^ mice at P22 and P35 were sectioned and examined by fluorescence microscope. DsRed-positive (Red) and DAPI-counterstained (blue) signals were shown. arrow: isthmus hyperplasia. (**E**) Quantification of Sox9-positive (Sox9^+^) cells in the bulge region from control and *Pofut1*/*Tgfb3*-Cre mice at P22. A bar diagram shows the number of Sox9^+^ cells per hair follicle (mean+/−s.d., n = 80) from two independent control and mutant pairs, **:P<0.01. Antibodies used are color-coded according to fluorophore-tagged secondary antibodies. DAPI counterstaining in blue. Scale bar, 50 µM.

Hair follicles of *Pofut1*/*Tgfb3*-Cre mice displayed disorganized bulge structure with isthmus hyperplasia at the first telogen (P22, Figure 4F), suggesting that Notch signaling loss changes the characteristics of bulge stem cells. Therefore, we performed double-staining for the basal keratinocyte marker K14 and bulge stem cell marker CD34 on control and mutant back skin samples at P22 (Figure 7A). The overall signal intensity of CD34 in mutant hair follicles was lower and more diffused than those of control hair follicles, while K14-expressing cell layers in mutant isthmus were thickened, suggesting a decrease of CD34-positive keratinocytes and a concomitant increase of K14-positive keratinocytes in the isthmus. In addition, back skin samples from control and mutant were immunostained for bulge/hair germ markers K15 [27] and Sox9 [28]. We found that K15 staining was diminished and scattered in mutant hair follicles, compared with a prominent K15 signal in the bulge region of control hair follicles at P22 (Figure 7B). Moreover, Sox9 staining and Sox9-positive cells in the bulge region of *Pofut1*/*Tgfb3*-Cre hair follicles were decreased at P22 (Figure 7C, E).

Bulge stem cells have been proposed to move down the hair follicle to renew hair follicle lineages, whereas they move upward to replenish the epidermis under stressed conditions. Therefore, we determined whether the isthmus hyperplasia seen in mutant hair follicles derives from *Pofut1*-deficient follicular keratinocytes using the G-Red allele, a Cre reporter that turns on DsRed expression upon Cre-mediated recombination. The back skin sections from the resulting control (*Pofut1* ^fx/wt^;G-Red^+/wt^;*Tgfb3*-Cre^+/wt^) and mutant (*Pofut1* ^fx/fx^;G-Red^+/wt^;*Tgfb3*-Cre^+/wt^) mice at P22 and P35 were examined by fluorescence microscopy (Figure 7D). At P22, *Pofut1*-deficient keratinocytes (DsRed-positive cells) populated the isthmus displaying hyperplasia morphology. Moreover, DsRed-positive signal was detected in the degenerated follicles and epithelial cysts at P35. Taken together, we conclude that isthmus hyperplasia and epithelial cysts derive from *Pofut1*-deficient follicular keratinocytes.

Since *Pofut1*-deficient hair follicles turned into epithelial cysts during the second hair cycle, immunohistochemical analyses were performed to determine whether abrogation of Notch signaling changes the cell fate choice of bulge stem cells. Staining of a panel of hair keratin markers revealed that follicular lineages were correctly specified but the IRS and hair shaft failed to complete their differentiation (Figure S5). Our finding essentially agrees with a notion that Notch signaling does not change the cell fate determination of hair follicle stem cells [15], [19], [26].

### Ablation of *Pofut1* in hair follicle lineages causes a delay in anagen re-entry

**Figure 8:**
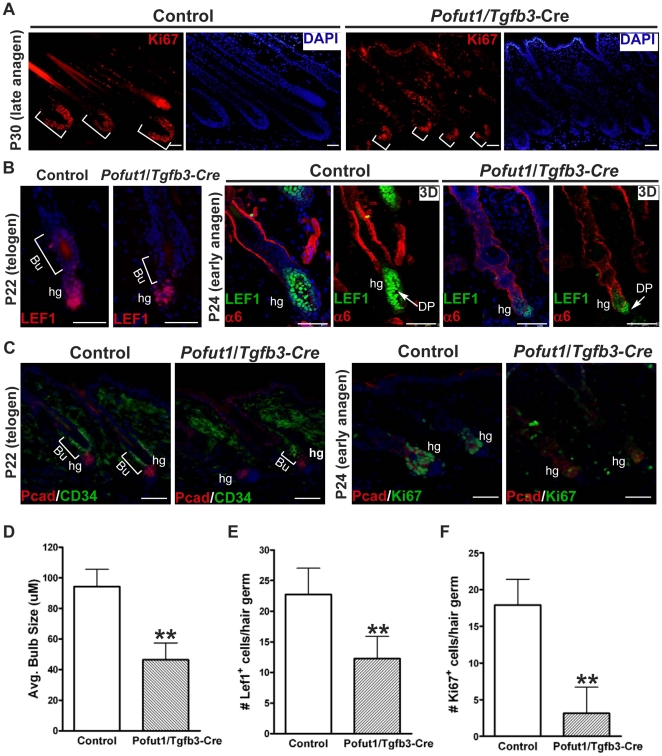
Inactivation of *Pofut1* in hair follicle lineages results in a defect in anagen re-entry. (**A**) Back skin samples from control and *Pofut1*/*Tgfb3*-Cre mice at P30 were immunostained for Ki67. bracket: proliferating (Ki67^+^) hair bulb matrix cells. (**B, C**) Back skin samples from control and *Pofut1*/*Tgfb3*-Cre mice at P22 (telogen) and P24 (early anagen) were immunostained for Lef1 (B, P22), double-stained for Lef1 and α6-integrin (α6) (B, P24), double-stained for P-cadherin (Pcad) and CD34 (C, P22), and double-stained for Pcad and Ki67 (C, P24). Confocal serial sections of Lef1 and α6 immunostaining were reconstructed to generate 3D images tilted at 45° on Z-axis (3D). (**D**) Quantification of the size of hair bulbs from control and *Pofut1*/*Tgfb3*-Cre mice at P30. A bar diagram shows the length of hair bulbs (mean+/−s.d., n = 30) from two independent control and mutant pairs, **: P<0.01. (**E, F**) Quantification of the Lef1-positive (Lef1^+^) and Ki67-positive (Ki67^+^) cells in the hair germ from control and *Pofut1*/*Tgfb3*-Cre mice at P24. Bar diagrams show the number of Lef1^+^ cells (E) and Ki67^+^cells (F) per hair germ (mean+/−s.d., n = 60) from two independent control and mutant pairs, **:P<0.01. Antibodies used are color-coded according to fluorophore-tagged secondary antibodies. DAPI counterstaining in blue. Bu: Bulge, hg: hair germ, DP: dermal papilla. Scale bar, 50 µM.

Since *Pofut1*/*Tgfb3*-Cre mice displayed smaller hair follicles than those of control mice at P30 (Figure 4G, H), back skin sections from control and mutant mice were immunostained for Ki67, a proliferating nuclear antigen, to examine the hair bulb (Figure 8A). We found a decrease in both the Ki67-positive matrix cells and the size of the hair bulb in mutant hair follicles at P30 (compares Ki67 and DAPI staining, Figure 8D). The size reduction of hair bulbs detected in mutant mice suggested an impairment of progenitor activation or an increase of cell apoptosis in the bulb matrix cells and was further investigated.

The hair germ, which is a distinct epithelial structure separating the bulge from the dermal papilla, directly contributes to the follicular lineages of the next hair cycle and was recently considered as an intermediate cell population between bulge stem cells and bulb transient amplifying cells [29]. To examine the status of hair germ between control and *Pofut1*/*Tgfb3*-Cre mice, back skin samples at P22 (telogen) and P24 (early anagen) were analyzed for hair germ markers (Lef1 and P-cadherin) and proliferation status. While the size of hair germs (Lef1^+^ and Pcad^+^ cells) was comparable between control and mutant mice at P22 (Figure 8B, C), mutant hair follicles displayed smaller hair germs with fewer Lef1^+^ positive cells than control hair follicles at P24 (Figure 8B, E). In addition, double staining for P-cadherin and Ki67 revealed a reduction in the numbers of Ki67^+^ cells in mutant hair germs compared to control hair germs at P24 (Figure 8C, F). These data collectively suggest that Notch signaling loss leads to a defect in anagen re-entry.

### *Pofut1*-deficient hair follicles display dysregulation of proliferation and apoptosis during the hair cycle transition

**Figure 9:**
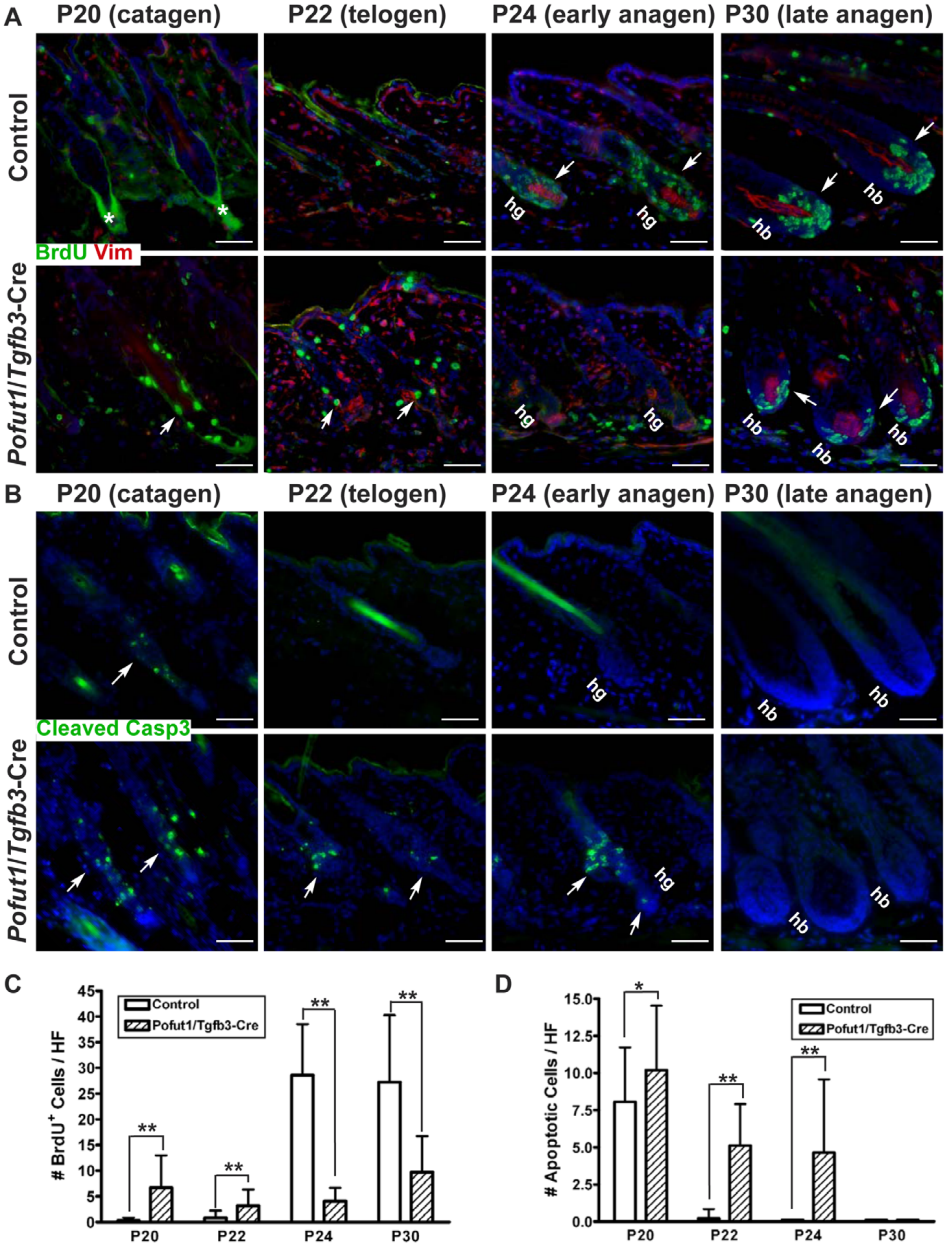
Disruption of *Pofut1* in hair follicle lineages results in dysregulation of proliferation and apoptosis during the hair cycle transition. (**A**) Control and *Pofut1*/*Tgfb3*-Cre mice were labeled with BrdU at P20 (catagen), P22 (telogen), P24 (early anagen), and P30 (late anagen). Back skin samples at stages indicated were double-stained for BrdU and vimentin. Immunostaining of vimentin was used to label the DP in sections and to exclude the misangled follicles in analyses. *: non-specific staining of the BrdU antibody. (**B**) Continuous serial sections from (A) were immunostained for cleaved Caspase-3. Positive signals are indicated by arrows. (**C**) Quantification of BrdU-positive (BrdU^+^) cells in (A). A bar diagram shows the number of BrdU^+^ cells per hair follicle (mean+/−s.d., n>40) at stages indicated from two to three independent control and mutant pairs. (**D**) Quantification of cells staining positive for cleaved Caspase3 in (B). A bar diagram shows the number of cleaved Caspase3-positive (apoptotic) cells per hair follicle (mean+/−s.d., n>40) at stages indicated from two to three independent control and mutant pairs, *: P<0.05, **:P<0.01. Antibodies used are color-coded according to fluorophore-tagged secondary antibodies. DAPI counterstaining in blue. hg: hair germ, hb: hair bulb. Scale bar, 50 µM.

Next, we performed cell proliferation and apoptosis assays to find a possible explanation for the bulge/hair germ phenotype seen in *Pofut1*/*Tgfb3*-Cre mice. Control and *Pofut1*/*Tgfb3*-Cre mouse pairs were BrdU-labeled at different hair cycle stages (from catagen to late anagen) and immunostained for BrdU (Figure 9A). Cell proliferation reflected as BrdU incorporation was detected in the hair germ at P24 (early anagen) and in the hair bulb at P30 (late anagen) in control samples as expected. In mutant hair follicles, BrdU staining was observed in the regressing ORS at P20 (catagen) as well as in the bulge/isthmus region at P22 (telogen). Strikingly, BrdU staining in the hair germ at P24 as well as in the hair bulb at P30 was largely reduced in mutant hair follicles, as revealed by quantitative analysis (Figure 9C).

The continuous serial sections used in the cell proliferation assay were also immunostained for cleaved Caspase-3, a known marker for cells committing apoptosis (Figure 9B). Cleaved Caspase 3-positive signals were detected only in the regressing ORS at P20 in control hair follicles, while positive signals can be detected in the regressing ORS at P20, as well as in the bulge/isthmus and hair germ region in mutant hair follicles at P22 and P24, as evidenced by quantitative analysis (Figure 9D). The absence of apoptosis in the *Pofut1*/*Tgfb3*-Cre hair bulb at P30 indicated that the reduction in the number of BrdU-positive cells detected in the mutant matrix cells is not due to increased apoptosis. Taken together, these data suggest that Notch signaling is essential for the homeostasis of bulge stem cells during the hair cycle transition.

### Abrogation of *Pofut1* in hair follicle lineages leads to DNA damage response and a paucity of DNA repair machinery

**Figure 10:**
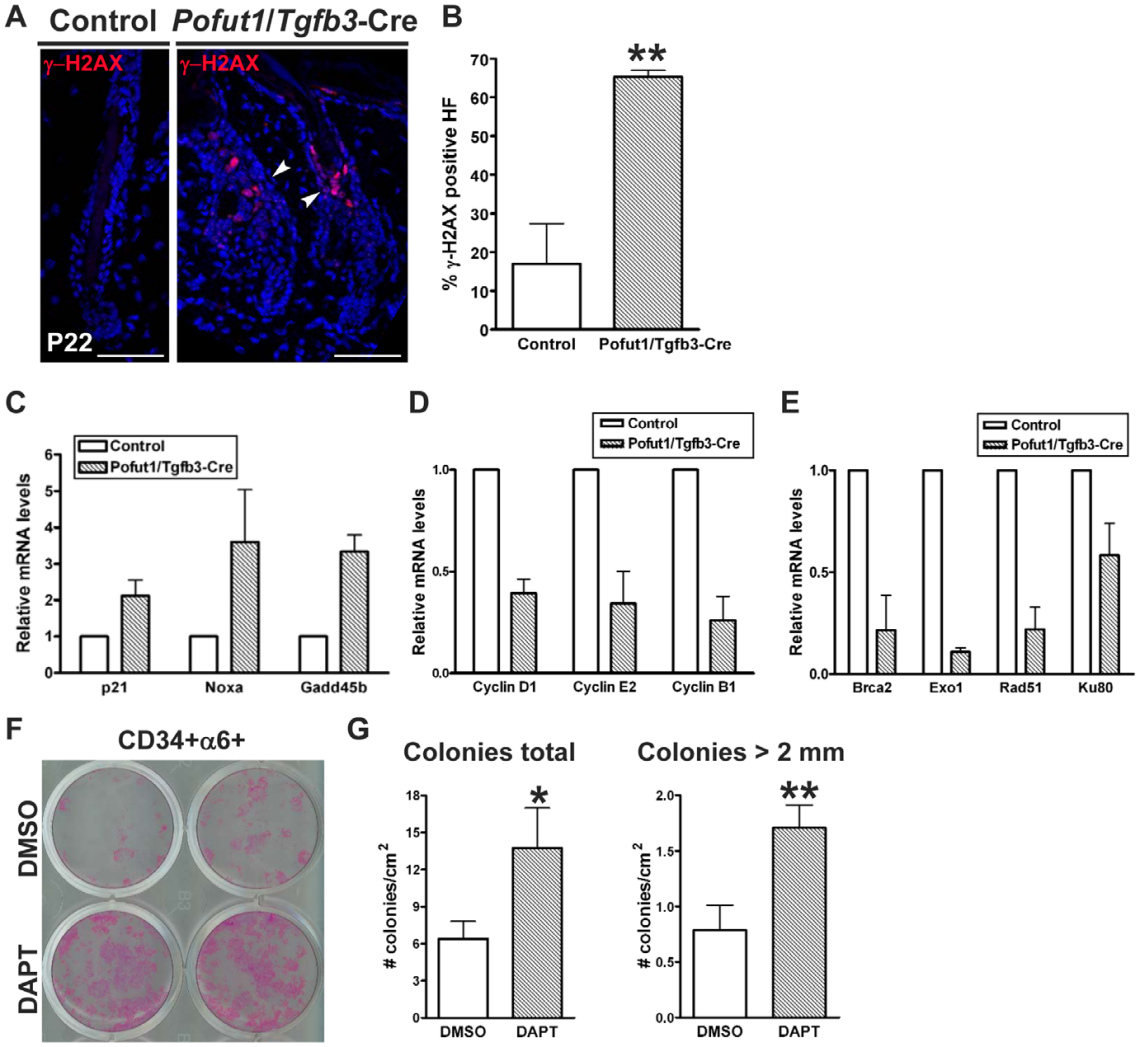
Ablation of *Pofut1* in hair follicle lineages induces DNA damage response and is possibly due to aberrant proliferation of bulge stem cells. (**A**) Back skin sections of control and *Pofut1*/*Tgfb3*-Cre mice at P22 were immunostained for γ-H2AX (arrowheads). Double-strand DNA-damage foci (γ-H2AX-positive) were detected in the nuclei of mutant follicular cells. (**B**) Quantification of γ-H2AX foci in hair follicles from control and *Pofut1*/*Tgfb3*-Cre mice. A bar diagram shows the percentage of γ-H2AX positive hair follicles (mean+/−s.d.) in over 50 fields at 20× magnification from three independent control and mutant pairs. **:P<0.01. (C–E) qRT-PCR analysis of a selected set of (**C**) p53 target genes, (**D**) cyclin genes, and (**E**) DNA repair genes on bulge keratinocytes isolated from control and *Pofut1*/*Tgfb3*-Cre mice. Bar diagrams show mRNA levels of genes indicated relative to their corresponding controls (mean+/−s.d., n>3). (**F**) Equal numbers of sorted CD34+α6+ keratinocytes from 8-week-old wild type mice were cultured with feeders and treated with either DMSO as a vehicle control or 10µM DAPT for 7 days. Representative culture plates with stained colonies are shown. (**G**) Quantification of the colony forming assay on sorted CD34+α6+ keratinocytes in the absence or presence of DAPT. Bar diagrams show the number of colonies per square cm (mean+/−s.d., n = 3) from counting total colonies generated and colonies larger than 2 millimeter, respectively.

The delayed anagen re-entry and dysregulation of proliferation/apoptosis during the hair cycle transition seen in *Pofut1*/*Tgfb3*-Cre mice prompted us to examine whether Notch signaling loss leads to DNA damage response (DDR) and cell senescence in mutant hair follicles. We could not detect cells in hair follicular lineages undertook cell senescence when assayed by the SAβ-gal staining (data not shown), while we observed an increase of DNA double-strand breaks (DSBs), as revealed by the presence of γ-H2AX foci, in *Pofut1*/*Tgfb3*-Cre follicular cells (Figure 10A, B). Next, we explored whether the DNA damage signal triggers the cell cycle checkpoint in mutant hair follicle stem cells. CD34^+^α6^+^ keratinocytes from control and *Pofut1*/*Tgfb3*-Cre mice at P22 were isolated by a FACS and analyzed for gene expression of a panel of pro-apoptotic genes, cell cycle regulators, and DNA repair genes by qRT-PCR (Figure 10C–E). We observed a modest increase of p21, and a greater increase of Noxa and Gadd45b expression levels in mutants. Correlating with the increased expression of p21 and Gadd45b, a decrease of CyclinD1, CyclinE2, and CyclinB1 expression levels was detected in mutants. Interestingly, we found downregulation of DNA repair genes such as Brca2, Rad51, Ku80, and Exo1 (homologous recombination and non-homologous end-joining pathways) in mutants [30]. These data suggest that Notch signaling loss in hair follicle lineages leads to increased expression of pro-apoptotic genes, induction of cell cycle checkpoints, and a paucity of DNA repair machinery.

Notch1 has been shown to function as a tumor suppressor in mouse skin [31] and *Pofut1*/*Tgfb3*-Cre mice displayed isthmus hyperplasia, suggesting that the DNA damage signal seen in *Pofut1*/*Tgfb3*-Cre hair follicles was induced by aberrant cell proliferation, as found in the case of oncogene-induced DNA damage checkpoint responses [32]. To test this hypothesis, bulge keratinocytes were isolated from 8 weeks old wild type mice by FACS and cultured in the absence or presence of a γ-secretase inhibitor DAPT. Significantly, we observed increases in both the numbers and the size of colonies in DAPT-treated bulge keratinocyte cultures when compared with DMSO-treated bulge keratinocyte cultures. Colonies in both cultures displayed holoclone morphology (data dot shown). Our data suggest that Notch signaling loss in the bulge stem cells results in aberrant cell proliferation and such a replication stress may induce DNA damage.

## Discussion

In our present study, we inactivated *Pofut1* and *Rbpj* in hair follicle lineages and the suprabasal layer of the epidermis using *Tgfb3*-Cre. We found that *Rbpj*/*Tgfb3*-Cre mutants displayed an earlier onset of epidermis and hair follicle defects compared to *Pofut1*/*Tgfb3*-Cre mutants. While *Pofut1*-deficient mouse embryos exhibited similar phenotype as *Rbpj*-deficient embryos [12], [33], postnatal phenotypes between *Pofut1* and *Rbpj* mutant mice have varied depending on the experimental context. During mammary gland development, defects were similar in *Pofut1* and *Rbpj* mutants, when genes were deleted using a MMTV-Cre driver line [34]. However, intestinal defects were less severe in *Pofut1*/*Villin*-Cre mutants than in comparable *Rbpj* mutants [35], [36]. Interestingly, in developing airways defects in Clara cells differentiation were similar between *Pofut1*/*Shh*-Cre and *Rbpj*/*Shh*-Cre mutants [37]; however, defects in neuroendocrine cells differentiation were more severe in *Pofut1* deletion than in *Rbpj* deletion and it was ascribed to a marginal reduction of *Hes1* expression by *Rbpj* deletion [38]. The delayed onset of epidermis and hair follicle defects seen in *Pofut1*/*Tgfb3*-Cre mutants may be due to less efficient deletion of *Pofut1* than *Rbpj* by *Tgfb3*-Cre or remaining Notch activity from Notch receptors in the absence of O-fucose modification [9]. We cannot exclude the possibility that Notch-independent effects of *Rbpj* [39], [40] may contribute to the severity of phenotypic changes observed in *Rbpj*/*Tgfb3*-Cre mice.

The obvious phenotypic difference between *Pofut1*/*Tgfb3*-Cre and *Rbpj*/*Tgfb3*-Cre mice is in sebaceous glands homeostasis. *Rbpj*/*Tgfb3*-Cre mice displayed sebaceous gland atrophy, consistent with a proposed role for Notch signaling in sebaceous gland development [15], [19]. Sebaceous glands in *Pofut1*/*Tgfb3*-Cre mice became disorganized and enlarged at the first telogen. While they seemed degenerated, they still appeared with sebum in the end of second hair cycle as evidenced by Oil Red O staining, suggesting that terminal differentiation of sebaceous glands into sebocytes is close to normal in *Pofut1*/*Tgfb3*-Cre mice. Given that *Tgfb3*-Cre induces recombination in the sebaceous glands in the Rosa26 reporter assay, Notch signaling may be required in the early step of sebaceous gland development but contributes less to homeostasis state. In support of this notion, Notch1-null embryonic stem cells are capable of differentiating into sebaceous glands in chimeric animals [41].

Embryonic inactivation of *Rbpj* in the basal keratinocytes using K14-Cre or Msx2-Cre resulted in a thinner skin with stratification defects at birth [15], [42], which leads to neonatal death or epidermal hyperplasia after birth depending on the severity of barrier function defects [15], [43]. Since *Tgfb3*-Cre induced gene recombination in the suprabasal layer of the epidermis, the reactive epidermal hyperplasia seen in *Rbpj*/*Tgfb3*-Cre and *Pofut1*/*Tgfb3*-Cre mice was less likely resulted from a deficiency in basal keratinocyte differentiation as demonstrated by histological and immunohistochemical analyses (Figure S2 and S4), but rather due to a defect in late-stage epidermal differentiation as evidenced by abnormal KGs distribution, decreased KG/LG volume density, and an increased level of monomeric filaggrin in the *Pofut1*/*Tgfb3*-Cre epidermis. Filaggrin monomer is required for keratin bundle formation and correct biogenesis of the stratum corneum, and its degradation and modification to form natural moisturizing factors in the stratum corneum is essential for maintaining epidermal hydration [44]. In support of the significance of our finding, several animal models with aberrant profilaggrin/filaggrin processing displayed compromised epidermal barrier function [45], [46], [47], and skin disorders in humans including ichthyosis vulgaris and atopic dermatitis are associated with null mutations within the *FLG* gene [44]. Our novel findings also complement gain of function studies in cell cultures and transgenic mice by demonstrating that Notch signaling participates in late-stage granular layer differentiation.

Hair follicle stem cells sit in the bulge region of hair follicles and switch between quiescence and proliferation states to maintain the hair cycle, and a delicate balance between theses two states is essential to preserve a functional stem cell population. *Pofut1*/*Tgfb3*-Cre hair follicles displayed aberrant telogen morphology at the first telogen and their bulge stem cell markers (CD34, K15, and Sox9) were diminished with a concomitant increase of K14-positive keratinocytes populating in the isthmus. Bulge stem cells may be less quiescent with Notch signaling loss, as evidenced by increased BrdU staining in *Pofut1*-deficient hair follicles from catagen to telogen, isthmus hyperplasia of *Pofut1*-deficient hair follicles in telogen, and increased colony forming ability of DAPT-treated bulge keratinocytes. Aberrant activation of bulge stem cells may create a replication stress that triggers DDR, and this notion is supported by several findings which correlate oncogene-induced aberrant DNA replication with DSB generation [48], [49], [50], [51]. The outcome of DDR is transient cell-cycle arrest and activation of DNA repair machinery, followed by either cellular senescence or apoptosis. Interestingly, our qRT-PCR analysis revealed an induction of pro-apoptotic genes and a decrease of DNA repair genes in *Pofut1*-deficient bulge keratinocytes, which may explain why we only detected apoptosis following the DDR in *Pofut1*/*Tgfb3*-Cre hair follicles.

In conclusion, we found that canonical Notch signaling is required for late-stage granular layer differentiation and correct filaggrin processing in the epidermis. Importantly, Notch signaling loss in hair follicle lineages leads to DNA damage response and loss of stem cell characteristics, which is possibly due to aberrant activation of bulge stem cells.

## Materials and Methods

### Mice, genotyping, timed pregnancy and embryo isolation

Generation of *Tgfb3-Cre* (*Tgfb3*-Cre^+/wt^), floxed *Pofut1* (*Pofut1* ^fx/fx^), and floxed *Rbpj* (*Rbpj* ^fx/fx^) mice has been described previously [23], [33], [52]. Rosa26 Cre reporter mice were obtained from the Jackson laboratory (Bar Harbor, ME, USA). G-Red mice were generated using a transgenic vector consisting of the cytomegalovirus (CMV) enhancer, the chicken β-actin promoter, a loxP cassette containing eGFP cDNA and SV40 polyA, and the downstream DsRedT1 cDNA [53] and SV40 polyA sequences. *Pofut1* ^fx/f*x*^ mice were crossed with *Tgfb3*-Cre mice to generate heterozygous *Tgfb3*-Cre^+/wt^;*Pofut1* ^fx/wt^ mice, which were then crossed with *Pofut1* ^fx/f*x*^ mice to create *Pofut1j* ^fx/f*x*^;*Tgfb3*-Cre conditional knockout mice (hereafter named *Pofut1*/*Tgfb3*-Cre). *Rbpj* conditional knockout mice (*Rbpj* ^fx/f*x*^;*Tgfb3*-Cre, hereafter named *Rbpj*/*Tgfb3*-Cre) were generated in the same way. Age-matched *Pofut1* ^fx/fx^ or *Pofut1* ^fx/wt^ mice, and *Rbpj* ^fx/fx^ or *Rbpj* ^fx/wt^ mice were used as littermate controls for *Pofut1* and *Rbpj* conditional knockout animals, respectively. Floxed *Pofut1* and G-Red mice were crossed to obtain mice harboring the genotype *Pofut1* ^fx/fx^;G-Red^+/wt^ for fate-mapping of *Pofut1*-deficient cells. All mice were maintained in mixed genetic backgrounds. To set up timed pregnancies, female mice were mated during the dark period of the controlled light cycle, and the day mice acquired vaginal plugs was designated as day 0.5. Genotyping was performed on tail biopsies by PCR. The primers for genotyping the floxed *Pofut1* and the floxed *Rbpj* alleles have been described elsewhere [52], [54]. For the Cre allele, the forward and reverse primers were 5′-GTCGATGCAACGAGTGATGAGG-3′ and 5′-TTCCATGTGAACGAACCTGGTCGA-3′, respectively. For the G-red allele, the forward and reverse primers were 5′-CTGCTAACCATGTTCATGCC-3′ and 5′-GTACTGGAACTGGGGGGACAG-3′, respectively.

### Ethics Statement

All animal work was conducted according to Taiwan COA national guidelines. All studies and procedures performed on mice were carried out at the research laboratory of the National Health Research Institutes (NHRI) and were approved by the NHRI Animal Care and Use Committee (NHRI-IACUC-097059 and 098050).

### Histological analysis and Immunostaining

Lower back skin samples were fixed with 4% paraformaldehyde for either 30 min on ice followed by processing for frozen sectioning, or for 4 h at room temperature followed by processing for paraffin sectioning. All samples were sectioned at 7 µm. For histology, sections were stained with Hematoxylin and Eosin using standard procedures. For immunostaining, fixed sections were blocked in blocking solution (5% normal goat serum, 2.5% bovine serum albumin, and 0.3% Triton-X 100 in PBS), and incubated with primary antibodies in blocking solution overnight at 4°C. After washing, sections were incubated with Dylight 488- or Dylight 594-conjugated secondary antibodies (Jackson ImmunoResearch) and counterstained with DAPI in mounting media (Vector Labs). Images were taken using an Olympus DP71 CCD device attached to an Olympus BX51 microscope with DP controller and DP manager software or using a Leica TCS SP5 confocal microscope system with Leica Power 3D software. Identical conditions of exposure and background balance for image capture were used for comparisons between control and mutant samples. Similar results were obtained with at least three independent sets of control and mutant pairs.

The sources and dilutions of primary antibodies were: Cre (1∶1000, Novagen), β-gal (1∶800, Cappel), Pofut1 (1∶100, Abcam), Hes1 (1∶100, Santa Cruz), K17 (1∶100, Lab Vision), K6 (1∶100, Lab Vision), AE15 (1∶100, Santa Cruz), AE13 (1∶100, Abcam), Gata3 (1∶150, Santa Cruz), Ki67 (1∶100, Lab Vision), α6-integrin (1∶100, eBioscience), laminin (1∶100, Lab Vision), CD34 (1∶100, eBioscience), K15 (1∶250, Lab Vision), Sox9 (1∶100, Santa Cruz), Lef1 (1∶1000, Cell Signaling), P-cadherin (1∶250, R&D), vimentin (1∶200, Abcam), K14 (1∶250, Lab Vision), K5 (1∶100, Abcam), K10 (1∶200, Lab Vision), loricrin (1∶500, Convance), filaggrin (1∶100 Convance), p63 (1∶200, Millipore), γ-H2AX (1∶600, Millipore), and TSLP (1∶100, R&D). For immunohistochemical analysis, sections were incubated with primary antibodies and then with biotinylated secondary antibody. Vectastain ABC Kit and DAB substrate Kit (Vector labs) were used to visualize the signal following the manufacturer's protocol. When staining with mouse monoclonal antibodies, sections were incubated with the unconjugated AffiniPure Fab fragment of goat anti-mouse IgG(H+L) (100 µg/ml, Jackson ImmunoResearch) for 1 h at room temperature to block endogenous mouse IgG.. When using biotin-conjugated secondary antibodies, endogenous biotin was blocked by incubating with 0.01% avidin in PBS for 15 min and then 0.005% biotin in PBS for another 15 min. Detailed protocols have been described by IHC world (http://www.ihcworld.com).

### Transmission electron microscopy and quantitative analysis

Tissue samples were prepared and embedded with the EMBed-812 embedding kit as previously described [55]. Quantitative data were obtained from two independent control littermate and mutant pairs. For each animal, 3 of the 10 tissue blocks of the skin were randomly chosen to be sectioned, and 10 to 15 sections were cut from each block. Twenty to 30 micrographs were randomly taken in the granular layer of the epidermis, and a total of 60 to 90 micrographs per area per animal were collected. The volume density expressed as the number of points overlying the keratohyalin granule/lamellar granule relative to the total number of points on the granular layer was obtained by the point-counting method of Weibel [56].

### R26R reporter assay, *In situ* hybridization, Oil Red O staining, Nile Red staining, and dye penetration assay

For the R26R reporter assay, embryos or frozen sections were fixed in 4% and 0.2% paraformaldehyde, respectively, on ice for 20 min and stained for β-galactosidase activity using X-gal as previously described [57]. Sections were counterstained with eosin after X-gal staining. RNA *in situ* hybridization was performed on 7 µM paraffin sections using digoxigenin-UTP-labeled antisense riboprobes. The hybridized probes were detected using alkaline phosphatase-conjugated anti-DIG sheep antibody and BM purple substrate (Roche) as previously described [58]. The *Tgfb3*, *Hes1*, *Hey1*, and *HeyL* riboprobes have been described elsewhere [59], [60], [61]. For Oil Red O staining, frozen sections were fixed in 4% paraformaldehyde on ice for 5 min and stained in 0.5% Oil Red O/100% propylene glycol in a 60°C oven for 8 min. After being rinsed in 85% propylene glycol, the slides were counterstained with hematoxylin and mounted in aqueous mounting medium (CC/mount, Sigma). Nile Red staining was done on frozen sections with 0.5 µg/ml Nile Red (Sigma) in 75% glycerol. For dye penetration assay, animals were submerged in an X-gal solution with a pH of 4.5 at 37°C for 16 hours as described [62].

### BrdU-labeling, cell proliferation, and apoptosis analyses

For BrdU labeling, mice were injected intraperitoneally with cell proliferation-labeling reagent (BrdU/FdU, Amersham) at 10 µl/g body weight and sacrificed 3 hours later. Cell proliferation analysis was done by immunostaining for BrdU incorporation using a Cell Proliferation kit (Amersham) following the manufacturer's protocol. For apoptosis analysis, frozen sections were fixed with 4% paraformaldehyde and immunostained with cleaved Caspase-3 antibody (1∶200, Cell Signaling). Positively stained cells were counted manually in a defined area of the tissues. Statistical analysis of cell counts in serial sections from two to three independent control and mutant pairs was performed using a Student's *t*-test, and a *P*-value less than 0.05 was considered to be significant.

### Western blotting, isolation of bulge keratinocytes by FACS, and *in vitro* colony forming assay

For Western blotting, the dorsal skin from control and mutant mice were treated with dispase (5 U/ml, Invitrogen) overnight at 4°C to isolate the epidermis, and followed by Trypsin-EDTA (0.25%, Invitrogen) treatment for 10 min at 37°C to dissociate the keratinocytes. After passing through a 70 µM cell strainer, primary keratinocytes were extracted with RIPA buffer plus protease inhibitor cocktail (Amresco) and phosphatase inhibitors (10 mM NaF, 1 mM Sodium Vanadate). Equal amounts of protein samples (30 µg of each) from control and mutant mice were used in SDS-PAGE and immunoblotted for filaggrin (1∶1000, Convance) and β-actin (1∶10000, Millipore) following the standard procedure. Isolation and culture of bulge keratinocytes based on α6-integrin and CD34 were performed as previously described [63]. Dorsal skin of mice in telogen (P21–P22, or 8 weeks old) was used for FACS on a FACS Aria machine (BD, Franklin Lakes, New Jersey, USA) and keratinocytes with high forward and side scatter as well as dead cells (7-AAD+) were gated out. For colony forming assay, sorted bulge keratinocytes were seeded at a density of 5000 cells per well in 12 well plates with mitotically arrested Swiss 3T3 cells, and cultures were maintained in E-media for 7–10 days. Cultures were gently treated with 0.25% Trypsin-EDTA to remove feeders, fixed with 4% paraformaldehyde, and stained with 0.5% Rhodamine B (Sigma).

### Total RNA isolation and quantitative real-time PCR (qRT-PCR)

The epidermis harvested from the dorsal skin by dispase was homogenized in TRIzol reagent (Invitrogen). Bulge keratinocytes isolated by a FACS were sorted directly into TRIzol reagent. Total RNA was isolated by the RNeasy Mini Kit (Qiagen) according to the manufacturer's protocol. The RNA quality and concentrations were measured by an Agilent 2100 Bioanalyzer (Agilent Technologies, Santa Clara, CA, USA). To obtain sufficient amount of samples for qRT-PCR analysis, total RNA isolated from bulge keratinocytes (∼200 ng from 10^5^ cells) was amplified by the RiboMultiplier sense-RNA Amplification Kit (Epicentre Biotech.). Equal RNA amounts (∼2 µg) were used to synthesize cDNA with the Transcriptor Reverse Transcriptase (Roche) and oligo-dT primers following the manufacturer's instructions. Real-time PCR was conducted using an ABI 7500 Real-Time PCR system (Applied Biosystems, Foster City, CA, USA) with FastStart Universal Probe Master (Rox, Roche) and probe/primer sets (Roche Universal Probe or Applied Biosystems Assay-on-Demand) designed to span over gene-specific exon-exon boundaries. Samples were analyzed by SDS Software v1.4 and normalized to the expression of the housekeeping gene ribosomal protein L7 (Rpl7) or hypoxanthine-guanine phosphoribosyltransferase (Hprt-1). The number of cycles needed to reach the crossing point (Ct) for each sample was used to perform the relative quantitative analysis with the 2^−^ΔΔCP method. Gene-specific universal probe numbers and primer sequences are available upon request.

## Supporting Information

Figure S1 Histological and immunofluorescence analysis of control and *Rbpj*/*Tgfb3*-Cre skin at P8. (A) Oil-Red O staining of back skin samples from control and *Rbpj*/*Tgfb3*-Cre mice. The sebaceous glands were regressed in the mutant mice. (B) Immunostaining for AE13 on back skin samples from control and *Rbpj*/*Tgfb3*-Cre mice. (C) Back skin sagittal sections from control and *Rbpj*/*Tgfb3*-Cre mice were immunostained for K10. Note *Rbpj*/*Tgfb3*-Cre skin does not display aberrant expression of K10 in the hair follicle lineages, a phenotype reported by *Rbpj* deletion in the DP. Antibodies used are color-coded according to fluorophore-tagged secondary antibodies. DAPI counterstaining in blue. Scale bar, 50 µM.
(TIF)

Figure S2 Histological and immunofluorescence analyses of control and *Rbpj*/*Tgfb3*-Cre skin at P1. (A) Back skin sagittal sections from control and *Rbpj*/*Tgfb3*-Cre mice were either H&E stained, or immunostained for K14, K10, loricrin (Lor), and Filaggrin (Filg). The expression levels of K14, K10, loricrin, and filaggrin are indistinguishable between control and mutant samples. (B) Nile Red staining of control and *Rbpj*/*Tgfb3*-Cre skin. Levels of the polar (orange) and neutral (green) lipids in the epidermis are comparable between control and mutant skin. (C) Dye penetration assay to examine the barrier function of control and *Rbpj*/*Tgfb3*-Cre mice. Antibodies used are color-coded according to fluorophore-tagged secondary antibodies. DAPI counterstaining in blue. Scale bar: 50 µM.
(TIF)

Figure S3 Notch signaling is dispensable for homeostasis of sebaceous glands. Oil Red O staining of back skin samples from control and *Pofut1*/*Tgfb3*-Cre mice at different time points (P14 to P58). Insets are magnified views of the sebaceous glands from the corresponding low magnification pictures. Scale bar, 50 µM.
(TIF)

Figure S4 Abrogation of *Pofut1* in the suprabasal layer of the epidermis leads to reactive epidermal hyperplasia. (A) Back skin samples from control and *Pofut1*/*Tgfb3*-Cre mice at P14 and P35 were either H&E stained or immunostained for filaggrin, loricrin, K10, and K14. The basement membrane appeared to be intact as revealed by α6-integrin immunostaining. Notably, an expansion of K14-expressing cell layers in the mutant epidermis at P35. (B) Back skin sections from control and *Pofut1*/*Tgfb3*-Cre mice at P35 were immunostained for K14 and loricrin (Lor), K10 and K5, K14 and Ki67, and P63. Notably, partial overlapping of K5 and K10 in the suprabasal layer (arrows) of the mutant epidermis at P35, indicating premature epidermal differentiation. Ki67-positive cells and p63-positive cells were detected in the suprabasal layer (arrows) of the mutant epidermis, indicating an epidermal hyperplasia. K6 and K17, two dysplasia markers of the epidermis, were not detected in the mutant interfollicular epidermis at P35 (data not shown). (C) The Nile Red staining revealed comparable lipid deposit in the stratum corneum of control and *Pofut1*/*Tgfb3*-Cre mice at P14. (D) Back skin sections of control and *Pofut1*/*Tgfb3*-Cre mice at P14 were immunostained for TSLP. The positive staining was detected in the suprabasal keratinocytes of the mutant epidermis. *: non-specific staining in the stratum corneum. Antibodies used are color-coded according to fluorophore-tagged secondary antibodies. DAPI counterstaining in blue. Scale bar: 50 µM. (E) qRT-PCR analysis of TSLP, S100a8, and S100a9 on back skin epithelium of control and *Pofut1*/*Tgfb3*-Cre mice at P14. Bar diagrams show mRNA levels of TSLP, S100a8, and S100a9 in *Pofut1*/*Tgfb3*-Cre epidermis relative to corresponding controls (mean+/−s.d., n = 3, three independent control and mutant pairs).
(TIF)

Figure S5 Abrogation of *Pofut1* in hair follicle lineages leads to hair follicle maturation defects. Immunostaining of a panel of hair keratin markers and Gata3 on back skin samples from control and *Pofut1*/*Tgfb3*-Cre mice at P15 and P30. The maturation defect of *Pofut1*/*Tgfb3*-Cre hair follicles was not due to a loss of contact between the hair bulb and its dermal papilla, as evidenced by double staining of AE15 and vimentin (Vim/AE15), and of AE13 and vimentin (Vim/AE13) in mutant hair follicles at P35 (insets in A). K17: ORS marker; K6: companion layer and medulla marker; AE15: IRS and medulla marker; AE13: hair shaft cuticle and cortex marker; vimentin: DP marker; Gata3: IRS marker. Antibodies used are color-coded according to fluorophore-tagged secondary antibodies as indicated. DAPI (nucleus) counterstaining in blue. Scale bar: 50 µM.
(TIF)
