# Supplementary figures and images for: Notch Signaling Regulates Late-Stage Epidermal Differentiation and Maintains Postnatal Hair Cycle Homeostasis
Source: PLoS One. 2011 Jan 18;6(1):e15842. doi: 10.1371/journal.pone.0015842 (PMC3022660; doi:10.1371/journal.pone.0015842)

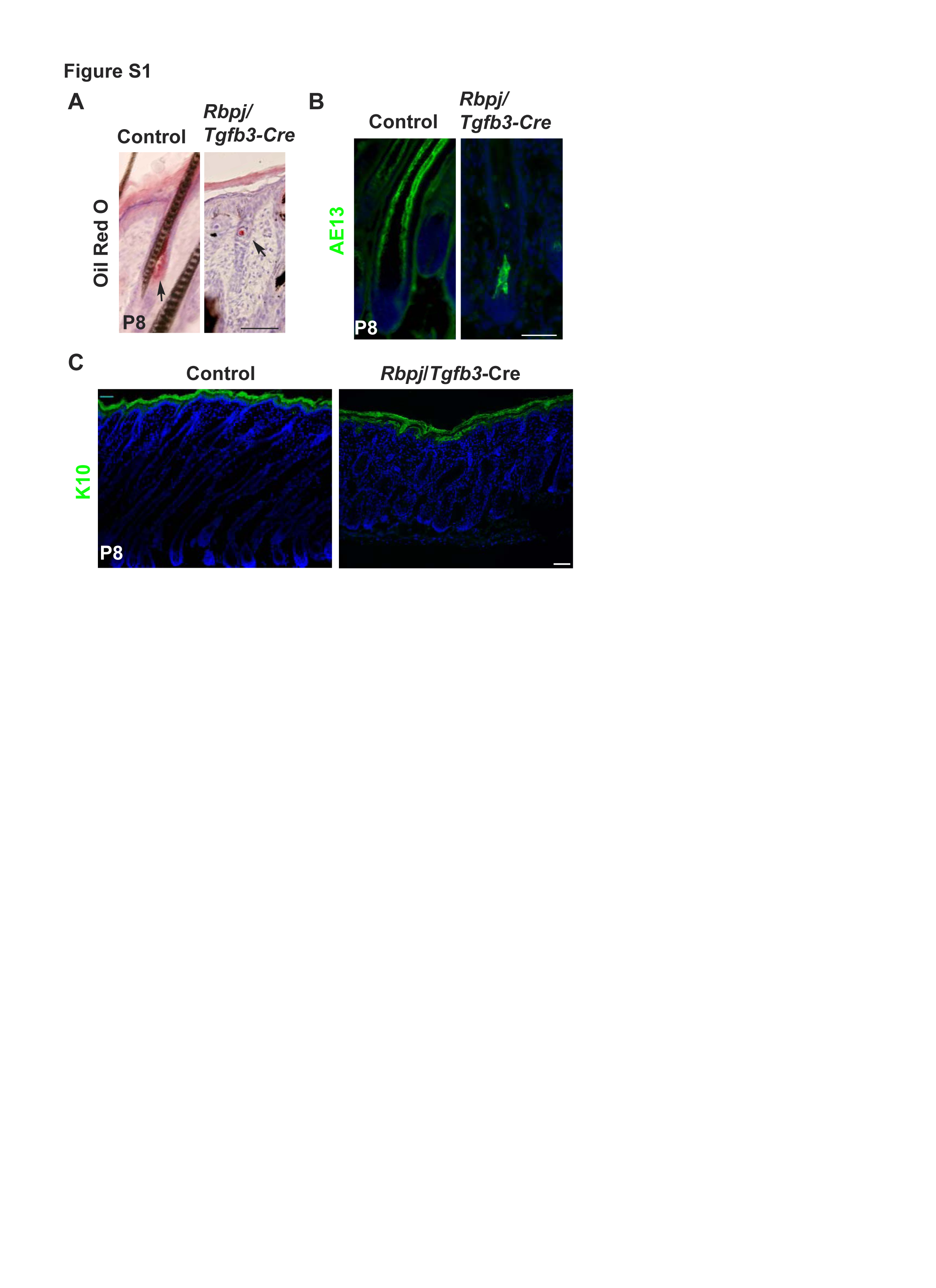

Supplement: Figure S1 — Histological and immunofluorescence analysis of control and Rbpj/Tgfb3-Cre skin at P8. (A) Oil-Red O staining of back skin samples from control and Rbpj/Tgfb3-Cre mice. The sebaceous glands were regressed in the mutant mice. (B) Immunostaining for AE13 on back skin samples from control and Rbpj/Tgfb3-Cre mice. (C) Back skin sagittal sections from control and Rbpj/Tgfb3-Cre mice were immunostained for K10. Note Rbpj/Tgfb3-Cre skin does not display aberrant expression of K10 in the hair follicle lineages, a phenotype reported by Rbpj deletion in the DP. Antibodies used are color-coded according to fluorophore-tagged secondary antibodies. DAPI counterstaining in blue. Scale bar, 50 µM. (TIF) [file pone.0015842.s001.tif]

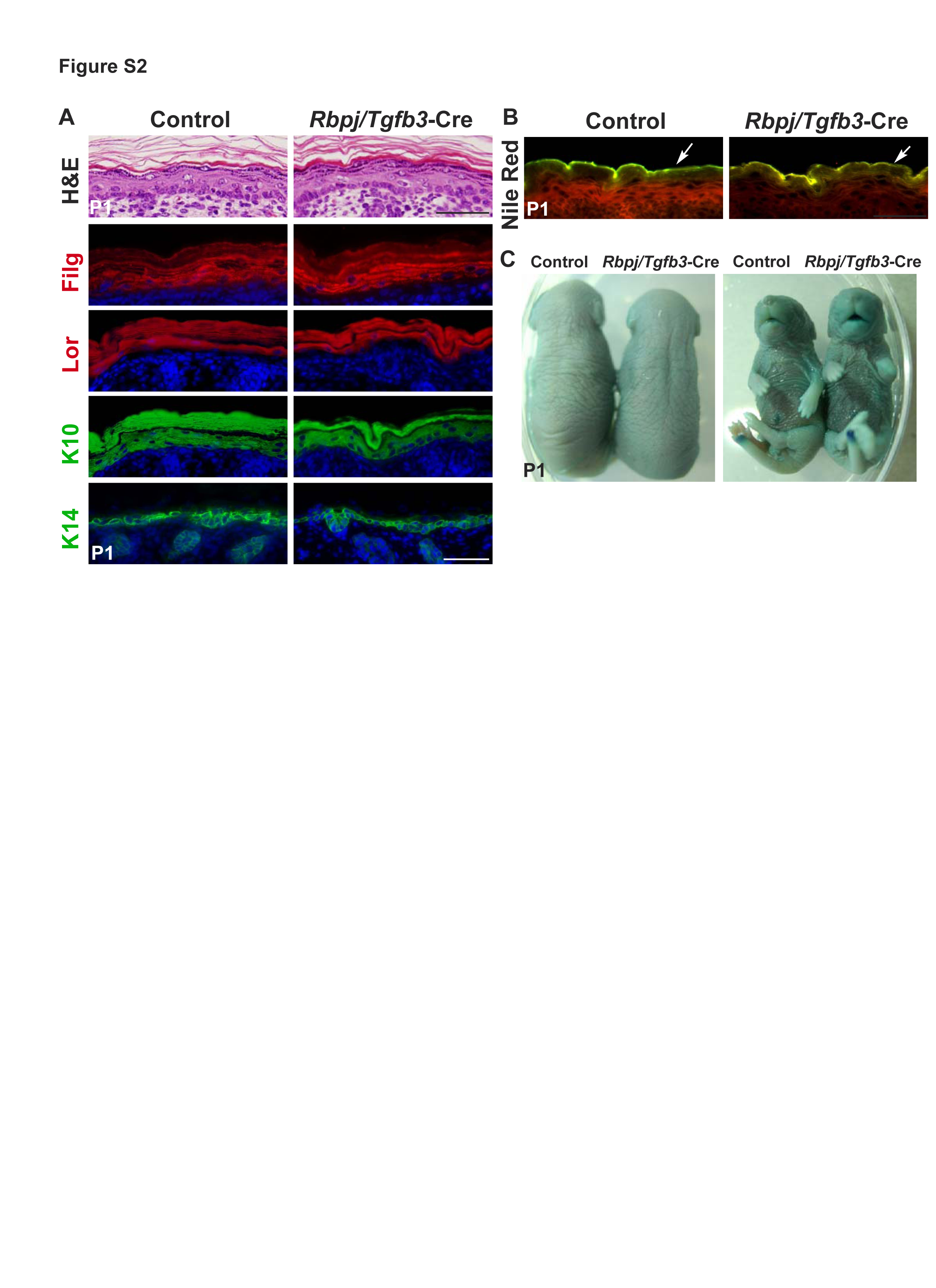

Supplement: Figure S2 — Histological and immunofluorescence analyses of control and Rbpj/Tgfb3-Cre skin at P1. (A) Back skin sagittal sections from control and Rbpj/Tgfb3-Cre mice were either H&E stained, or immunostained for K14, K10, loricrin (Lor), and Filaggrin (Filg). The expression levels of K14, K10, loricrin, and filaggrin are indistinguishable between control and mutant samples. (B) Nile Red staining of control and Rbpj/Tgfb3-Cre skin. Levels of the polar (orange) and neutral (green) lipids in the epidermis are comparable between control and mutant skin. (C) Dye penetration assay to examine the barrier function of control and Rbpj/Tgfb3-Cre mice. Antibodies used are color-coded according to fluorophore-tagged secondary antibodies. DAPI counterstaining in blue. Scale bar: 50 µM. (TIF) [file pone.0015842.s002.tif]

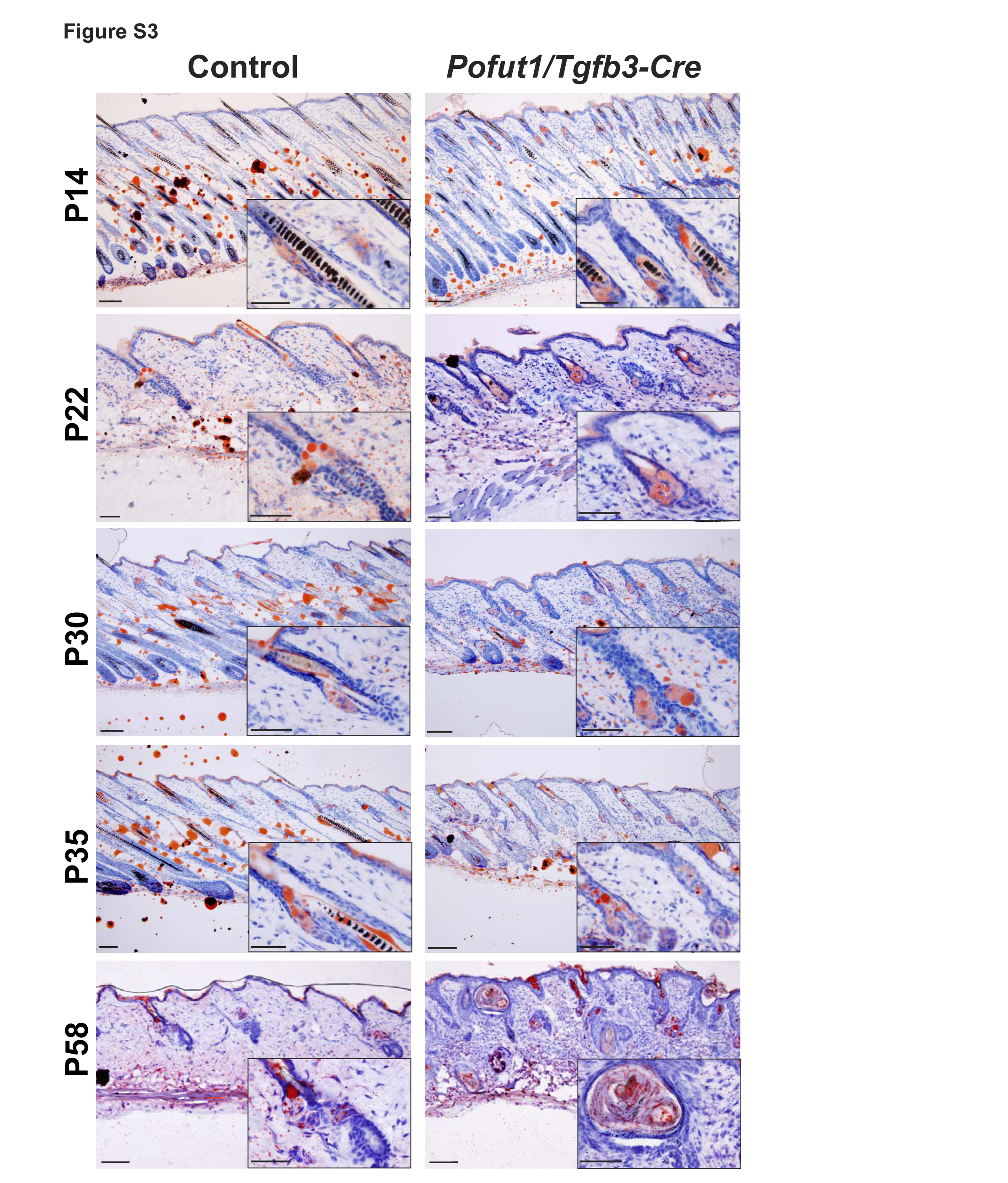

Supplement: Figure S3 — Notch signaling is dispensable for homeostasis of sebaceous glands. Oil Red O staining of back skin samples from control and Pofut1/Tgfb3-Cre mice at different time points (P14 to P58). Insets are magnified views of the sebaceous glands from the corresponding low magnification pictures. Scale bar, 50 µM. (TIF) [file pone.0015842.s003.tif]

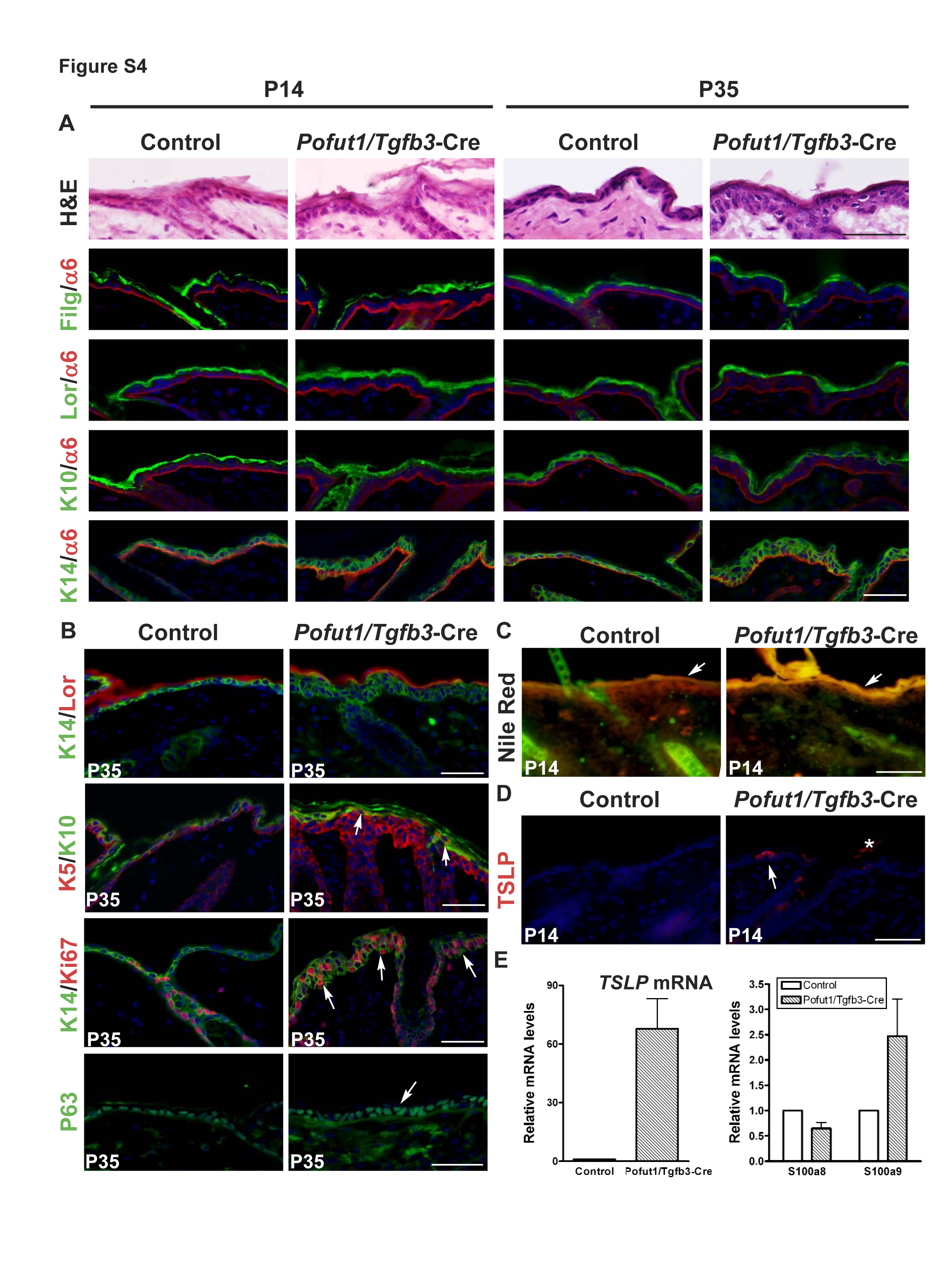

Supplement: Figure S4 — Abrogation of Pofut1 in the suprabasal layer of the epidermis leads to reactive epidermal hyperplasia. (A) Back skin samples from control and Pofut1/Tgfb3-Cre mice at P14 and P35 were either H&E stained or immunostained for filaggrin, loricrin, K10, and K14. The basement membrane appeared to be intact as revealed by α6-integrin immunostaining. Notably, an expansion of K14-expressing cell layers in the mutant epidermis at P35. (B) Back skin sections from control and Pofut1/Tgfb3-Cre mice at P35 were immunostained for K14 and loricrin (Lor), K10 and K5, K14 and Ki67, and P63. Notably, partial overlapping of K5 and K10 in the suprabasal layer (arrows) of the mutant epidermis at P35, indicating premature epidermal differentiation. Ki67-positive cells and p63-positive cells were detected in the suprabasal layer (arrows) of the mutant epidermis, indicating an epidermal hyperplasia. K6 and K17, two dysplasia markers of the epidermis, were not detected in the mutant interfollicular epidermis at P35 (data not shown). (C) The Nile Red staining revealed comparable lipid deposit in the stratum corneum of control and Pofut1/Tgfb3-Cre mice at P14. (D) Back skin sections of control and Pofut1/Tgfb3-Cre mice at P14 were immunostained for TSLP. The positive staining was detected in the suprabasal keratinocytes of the mutant epidermis. *: non-specific staining in the stratum corneum. Antibodies used are color-coded according to fluorophore-tagged secondary antibodies. DAPI counterstaining in blue. Scale bar: 50 µM. (E) qRT-PCR analysis of TSLP, S100a8, and S100a9 on back skin epithelium of control and Pofut1/Tgfb3-Cre mice at P14. Bar diagrams show mRNA levels of TSLP, S100a8, and S100a9 in Pofut1/Tgfb3-Cre epidermis relative to corresponding controls (mean+/−s.d., n = 3, three independent control and mutant pairs). (TIF) [file pone.0015842.s004.tif]

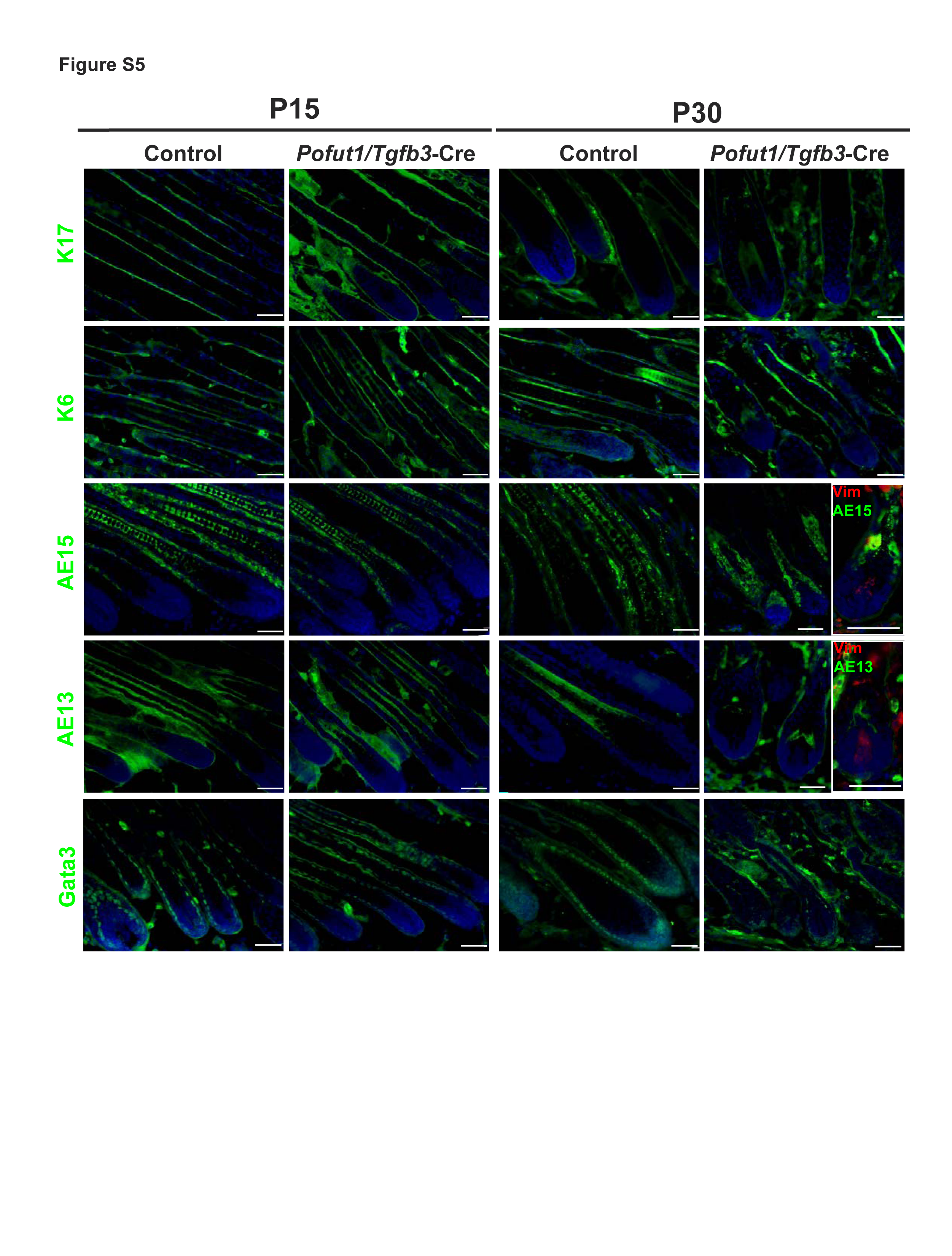

Supplement: Figure S5 — Abrogation of Pofut1 in hair follicle lineages leads to hair follicle maturation defects. Immunostaining of a panel of hair keratin markers and Gata3 on back skin samples from control and Pofut1/Tgfb3-Cre mice at P15 and P30. The maturation defect of Pofut1/Tgfb3-Cre hair follicles was not due to a loss of contact between the hair bulb and its dermal papilla, as evidenced by double staining of AE15 and vimentin (Vim/AE15), and of AE13 and vimentin (Vim/AE13) in mutant hair follicles at P35 (insets in A). K17: ORS marker; K6: companion layer and medulla marker; AE15: IRS and medulla marker; AE13: hair shaft cuticle and cortex marker; vimentin: DP marker; Gata3: IRS marker. Antibodies used are color-coded according to fluorophore-tagged secondary antibodies as indicated. DAPI (nucleus) counterstaining in blue. Scale bar: 50 µM. (TIF) [file pone.0015842.s005.tif]
